# Application of Virtual Twin PBPK Models in Individuals with Obesity via CYP3A4 Phenotyping Using Endogenous Biomarker Data

**DOI:** 10.1002/cpt.70369

**Published:** 2026-06-18

**Authors:** Nihan Izat, Haribhau Kangne, Rasmus Jansson‐Löfmark, Jens K. Hertel, Amin Rostami‐Hodjegan, Ida Robertsen, Aleksandra Galetin

**Affiliations:** ^1^ Centre for Applied Pharmacokinetic Research, The University of Manchester Manchester UK; ^2^ Drug Metabolism and Pharmacokinetics, Research and Early Development, Cardiovascular, Renal and Metabolism (CVRM), BioPharmaceuticals R&D, AstraZeneca Gothenburg Sweden; ^3^ Department of Endocrinology, Obesity and Nutrition Vestfold Hospital Trust Tønsberg Norway; ^4^ Certara Predictive Technologies Sheffield UK; ^5^ Section for Pharmacology and Pharmaceutical Biosciences, Department of Pharmacy University of Oslo Oslo Norway

## Abstract

Plasma 4β‐hydroxycholesterol (4β‐OHC) normalized for the levels of its parent cholesterol (4β‐OHC/C) is an endogenous biomarker for hepatic CYP3A4. This study evaluated CYP3A4 activity longitudinally in individuals while their obesity status was changing. 4β‐OHC/C was measured pre‐surgery (*n* = 54) and 2 years after Roux‐en‐Y gastric bypass (*n* = 30) and used as an input for creating virtual twins of each patient within physiologically‐based pharmacokinetic (VT‐PBPK) models, alongside other available data (e.g., demographics). Additionally, individual CYP3A4 abundance quantified from liver biopsies during surgery was available. Predicted CYP3A4 abundance using 4β‐OHC/C was within twofold of the measured abundance in 85% of the individuals (*n* = 54). Pre‐surgery VT‐PBPK models informed by biomarker data predicted area under curve after intravenous midazolam (AUC_inf,iv_) within twofold of observed values in 85% of individuals (geometric mean fold error (GMFE) = 1.58). VT‐PBPK models using CYP3A4 protein measurements showed similar performance (83% within twofold, GMFE = 1.66), verifying the biomarker approach. The post‐surgery VT‐PBPK models were revised to reflect surgery‐related alterations and changes in obesity status. Hepatic CYP3A4 activity at 2 years was predicted from 4β‐OHC/C measured in the same individuals. Biomarker‐informed models predicted midazolam AUC_inf,iv_ within twofold for 87% of individuals (GMFE = 1.48; *n* = 30). ~60% of observed oral midazolam PK parameters were predicted within twofold, improving to 70% when the surgery model assumed full intestinal CYP3A4/5 recovery. This study highlights the utility of 4β‐OHC/C for continuous monitoring of CYP3A4 activity and possibility to use biomarker‐informed VT‐PBPK models for individual dose requirements of intravenously administered CYP3A substrates in individuals with obesity over the course of weight loss treatment.

**Study Highlights**

**WHAT IS THE CURRENT KNOWLEDGE ON THE TOPIC?**

The virtual twin physiologically‐based pharmacokinetic (VT‐PBPK) modeling is an emerging tool for model‐informed precision dosing. Obtaining individual data on abundance of drug‐metabolizing enzymes and transporters remains challenging due to limited availability of tissue proteomic data in patients. The plasma 4β‐hydroxycholesterol/cholesterol ratio (4β‐OHC/C) reflects steady‐state activity of hepatic CYP3A4, which may vary over time in patients with obesity due to heterogeneous weight loss following diet, pharmacotherapy, or surgery.

**WHAT QUESTION DID THIS STUDY ADDRESS?**

Can 4β‐OHC/C be used as an endogenous biomarker to monitor weight loss‐associated changes in individual hepatic CYP3A4 activity?

**WHAT DOES THIS STUDY ADD TO OUR KNOWLEDGE?**

Biomarker‐informed VT‐PBPK modeling enabled subject‐specific longitudinal characterization of CYP3A4 activity in patients with obesity and prediction of midazolam PK in the same individuals before and after gastric bypass surgery.

**HOW MIGHT THIS CHANGE CLINICAL PHARMACOLOGY OR TRANSLATIONAL SCIENCE?**

Biomarker‐informed VT‐PBPK modeling can be used to assess dynamic, individual‐level changes in hepatic CYP3A4‐mediated metabolism. It can support drug development and precision dosing, with applicability to other special populations in which CYP3A4 expression is altered.

The virtual twin approach linked to physiologically‐based pharmacokinetic modeling (VT‐PBPK) is an emerging tool for predicting individual drug exposure. VT‐PBPK models incorporate subject‐specific data, such as patient demographics, plasma protein levels, individual enzyme/transporter abundance, and routinely collected physiological data (e.g., organ function).^1^, ^2^ So far, the majority of VT‐PBPK models have primarily focused on substrates of cytochrome P450 (CYP450) enzymes and have relied on either genotyping or phenotyping to virtualize individual CYP activity. For example, paraxanthine‐to‐caffeine ratio in saliva^3^ or plasma^4^ has been used to individualize CYP1A2 activity in VT‐PBPK models for the prediction of olanzapine^3^ or caffeine^4^ exposure, respectively. Alternatively, CYP1A2 genotyping, with adjustment for smoking status, has been employed to predict clozapine pharmacokinetics (PK).^5^, ^6^ Genotyping alone is not expected to fully account for variability in enzyme/transporter activity across patients; it may also be confounded by transient physiological states and drug–drug interactions (DDIs).^7^ Recently, the 1′‐hydroxymidazolam/midazolam ratio was used for CYP3A4 phenotyping,^1^ alongside P‐gp phenotyping based on fexofenadine. The added value of individual CYP3A and P‐gp phenotypes in VT‐PBPK models for apixaban and rivaroxaban was most evident in patients at higher bleeding risk.

Recently, we developed VT‐PBPK models for individuals with obesity using their actual hepatic enzyme and transporter abundance measurements.^2^ However, the tissue biopsy sampling and subsequent proteomics analyses are often challenging to obtain from patients. Therefore, novel tools such as liquid biopsy of plasma exosomes may provide a non‐invasive alternative for assessing individual abundance of relevant enzymes/transporters. Recently, liquid biopsy‐derived CYP3A4 abundance data were used to individualize VT‐PBPK models, leading to a more precise prediction of midazolam PK in patients with renal impairment.^8^ However, continuous monitoring of dynamic changes in enzyme/transporter activity at multiple time points may require more high‐throughput and cost‐effective characterization methods such as endogenous biomarker measurements.^9^, ^10^

4β‐hydroxycholesterol (4β‐OHC) is a cholesterol metabolite formed mainly via CYP3A4, with CYP3A5 and CYP3A7 contributing only 5.6% and 2.8% relative to CYP3A4, respectively.^11^, ^12^ 4β‐OHC is used as an endogenous plasma biomarker of hepatic CYP3A4 activity,^10^, ^12^, ^13^ reflecting steady‐state changes in enzyme activity due to its long half‐life (60 hours to 17 days).^14^ With the recently developed fit‐for‐purpose 4β‐OHC PBPK model that successfully predicted hepatic CYP3A induction,^15^ clinical drug development is expected to make greater use of this biomarker. The current evidence of 4β‐OHC reflecting disease‐mediated changes in CYP3A activity is limited to reports in patients with rheumatoid arthritis^16^ and obesity.^17^ Based on kinetic principles, 4β‐OHC should be normalized relative to its parent cholesterol (4β‐OHC/C),^18^ so that the measurement reflects metabolic activity rather than baseline differences in precursor levels.^19^, ^20^, ^21^ The individual variation in cholesterol is smaller than the variation in metabolic activity (i.e., formation is less variable than elimination) and cholesterol levels explain only 9% of 4β‐OHC variability.^22^ Nevertheless, the 4β‐OHC/C is the more kinetically appropriate measure, especially in populations with abnormal cholesterol levels (patients taking antiretrovirals)^20^ and individuals with metabolic disorders.^21^

Obesity is a complex metabolic disease defined by a body mass index (BMI) of 30 kg/m^2^ or greater, and associated with anatomical, physiological, and biological changes that affect drug pharmacokinetics.^23^ Negative correlations were observed between BMI and 4β‐OHC concentrations in individuals with normal weight to severe obesity (BMI 18–63 kg/m^2^).^17^ These findings were consistent with significantly inverse correlations between the BMI and CYP3A4 abundance in the liver,^24^ emphasizing the relevance of assessing 4β‐OHC in obesity. Moreover, individuals with obesity may experience different extents of weight loss following diet, drug treatment or surgery, which may lead to variable physiological changes and corresponding effects on CYP3A4 activity over time.

The present study aimed to develop VT‐PBPK models for individuals with different stages of obesity (before and after gastric bypass surgery) using the endogenous CYP3A biomarker 4β‐OHC/C for CYP3A phenotyping. First, we compared the performance of biomarker‐informed VT‐PBPK models with those incorporating directly measured hepatic CYP3A4 abundance, using midazolam as a probe substrate. Subsequently, the biomarker‐informed models were applied to predict CYP3A‐mediated changes in midazolam PK 2 years after gastric bypass surgery, assessing the impact of weight loss and physiological changes on both the biomarker and midazolam exposure.

## METHODS

### Clinical data

Participants with/without obesity from the COCKTAIL study (NCT02386917)^25^ were subject to VT‐PBPK model simulations. Individuals who had undergone Roux‐en‐Y gastric bypass (RYGB) surgery (*n* = 36) and individuals who had undergone cholecystectomy (*n* = 18) were included for the construction of pre‐surgery VTs considering data availability. Pretreatment 4β‐OHC data available for individuals in the diet group of the COCKTAIL study (*n* = 40) were also considered for the investigation of BMI and 4β‐OHC relationship. At the end of year 2, 30 individuals of the initial RYGB cohort were included in VT‐PBPK simulations due to data availability. Medications, including statins, and/or other substances known to affect CYP3A activity were discontinued for at least seven half‐lives prior to the PK investigational day, and none of the patients used CYP3A4 inducers or time‐dependent inhibitors. The details of the PK study,^25^ quantification of 4β‐OHC, cholesterol,^17^ and midazolam^26^ concentrations, CYP3A5 genotyping,^17^ and proteomics assays on liver tissue biopsies collected during surgeries^27^ have been published previously. Further details are in the Suppl. methods Data S1.

Non‐compartmental PK analysis of midazolam PK following semi‐simultaneous oral (1.5 mg) and intravenous (iv) dosing (1 mg at 4 hours) was performed using the “NonCompart” package in RStudio (version 2025.09.2 + 418, Posit Software, Boston, MA, USA). Due to staggered dosing, the area under the plasma concentration–time curve extrapolated to infinity after the iv dose (AUC_inf,iv_) was obtained by subtracting the oral AUC extrapolated from 0 to 4 hours (AUC_inf,oral_) from the total AUC extrapolated to infinity (AUC_inf_) (Eq. 1). Weight‐normalized systemic clearance was calculated using Eq. 2 after unit conversions.

(1) $${\mathrm{AUC}}_{\inf,\mathrm{iv}}\;\left(\mathrm{ng}.h/\mathrm{mL}\right)={\mathrm{AUC}}_{\inf}-{\mathrm{AUC}}_{\inf,\text{oral}}$$

(2) $${\mathrm{CL}}_{\mathrm{iv}}\;\left(L/h/\mathrm{kg}\right)=\frac{{\text{Dose}}_{\mathrm{iv}}/{\mathrm{AUC}}_{\inf,\mathrm{iv}}}{\text{Body weight}}$$

### Database analysis

A literature search of reported 4β‐OHC and 4β‐OHC/C data in healthy adults was performed. Data were excluded if the BMI range was not reported. Literature data from Asian ethnicity were excluded considering the COCKTAIL study population (99% white).^26^ When 4β‐OHC/C data were not available, individual or mean 4β‐OHC concentrations were used after normalizing with the mean total cholesterol measured in the same samples. Collated data were merged with the 4β‐OHC/C data from the COCKTAIL trial (before surgery/diet treatment). Weighted mean of 4β‐OHC/C from individuals with BMI range between 18 and 30 kg/m^2^ was used as the healthy reference biomarker level.

### Development of virtual twins

**Figure 1:**
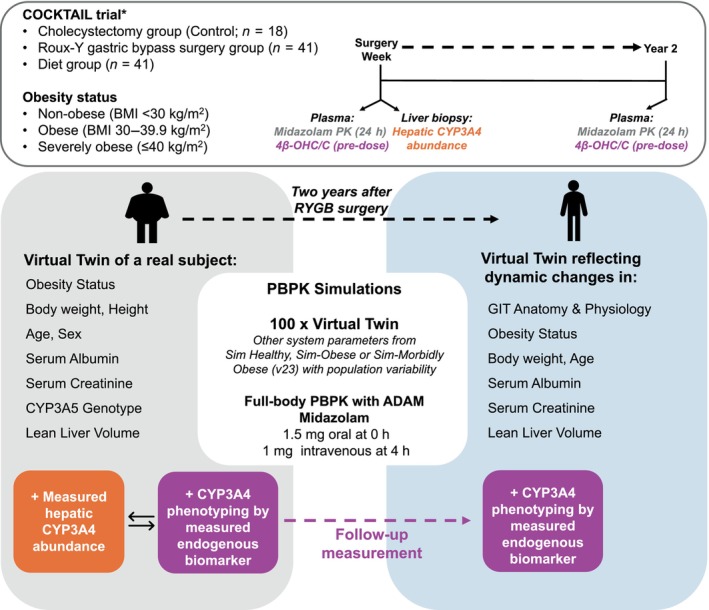
Virtual twin PBPK model framework applied for COCKTAIL trial^25^ participants with no obesity, obesity, and severe obesity before and after Roux‐en‐Y gastric bypass (RYGB) surgery informed with subject‐specific data measured at both time points. 24‐hour pharmacokinetic (PK) study for midazolam was done 1 day before the surgery. Sample collected at time zero (pre‐treatment) was used to quantify biomarker baseline levels (4β‐hydroxycholesterol/cholesterol ratio). *Further details of the clinical study have been published previously.^25^

Virtual twin PBPK models were developed in Simcyp PBPK Simulator (version 23) and simulations were performed with The Simcyp R package version 23.0.64 (Certara, Sheffield, UK) (**Figure** **1**). The Simcyp v23 library population of Sim‐Healthy Volunteers was used for individuals with a BMI < 30 kg/m^2^ (non‐obese). The “Sim‐Obese” and “Sim‐Morbidly Obese” populations were used for individuals with a BMI of 30–39.9 kg/m^2^ and ≥ 40 kg/m^2^, respectively. Each patient was matched to their known demographics; measured covariates are listed in **Figure** **1**. Lean liver volume for each individual was predicted using measured fat‐free mass.^2^, ^28^

CYP3A4 abundance was individualized using either the actual protein abundance (based on the sample at surgery) or CYP3A4 phenotyping based on 4β‐OHC/C data (at both surgery week and 2 years after) using Eq. 3. The CV in hepatic CYP3A4 abundance was set to zero.

(3) $${\left[\mathrm{CYP}3A4\right]}_{i}\;\left(\text{pmol}/\mathrm{mg}\;\text{microsomes}\right)={\left[\mathrm{CYP}3A4\right]}_{\text{healthy}}\times \frac{{\left[4\text{βOHC}/C\right]}_{i}}{{\left[4\text{βOHC}/C\right]}_{\text{healthy}}}$$

where [CYP3A4]_i_ and [4βOHC/C]_i_ refer to the hepatic abundance and biomarker level in each individual i. [CYP3A4]_healthy_ was set at 137 pmol/mg microsomal protein (Simcyp v23 built‐in value via meta‐analysis) as healthy population reference. [4βOHC/C]_healthy_ was obtained from individuals without obesity via literature analysis.

Individuals with CYP3A5 *1/*3 and *3/*3 genotypes were identified as extensive metabolizers (EMs) and poor metabolizers (PMs), respectively. Simulator's CYP3A4‐CYP3A5 correlation was activated to estimate CYP3A5 abundance in the liver for CYP3A5 EMs. CYP3A5 abundance was set to zero for PMs. The impact of CYP3A5 was minimal since the study group consisted mostly of PMs.

Two years post‐surgery, real life changes including altered gastrointestinal tract physiology and reduced body weight were used to revise VT models (**Figure** **1**). Advanced Dissolution Absorption and Metabolism (ADAM) was altered based on prior work^29^, ^30^ and adjusted to reflect the current RYGB surgery measurements of biliopancreatic (60 cm) and alimentary limbs (120 cm).^25^ Details of all modified system parameters related to RYGB surgery are provided in **Table** **S1**. RYGB‐based modifications of VT‐PBPK models resulted in reduced total intestinal CYP3A4 abundance (from 65.4 to 45 nmol/total gut) and CYP3A5 abundance (from 23.3 to 16 nmol/total gut). An alternative scenario with full recovery of intestinal CYP3A4/5 abundance to pre‐surgery values, with higher absolute enzyme abundances in the remaining intestinal regions, was also explored in simulations.

Midazolam full‐body PBPK with ADAM model (**Table** **S2**) was validated using independent clinical datasets after oral administration of midazolam in Sim‐healthy, obese, and RYGB surgery populations with matching trial designs (**Table** **S3**) before its use in VT‐PBPK simulations. Each VT was replicated 100 times in the trial design to account for variability and uncertainty in the system parameters for which individual data were not available. Midazolam dose and simulation time matched the clinical study design of the COCKTAIL trial.

### Statistical analysis and assessment of model performances

Spearman rank correlations between the 4β‐OHC/C, hepatic CYP3A4 abundance, and weight‐normalized systemic clearance of midazolam at the week of surgery were assessed using R stats package.^31^ The predictive performance of VT‐PBPK model simulations was assessed by comparing the individual observed mean of plasma concentration data with the mean, 5th, and 95th percentiles of predictions. Overall model performances were evaluated via average fold error (AFE), geometric mean fold error (GMFE), and the root mean squared error (RMSE)^32^ of the predicted vs. observed PK parameters across all individuals after iv and oral midazolam administration before and after RYGB surgery (Eqs. (4), (5), (6)).

(4) $$\mathrm{AFE}=10^{\frac{1}{n}\sum \log\left(\frac{\text{Predicted}}{\text{Observed}}\right)}$$

(5) $$\mathrm{GMFE}=10^{\frac{1}{n}\sum |\log\left(\frac{\text{Predicted}}{\text{Observed}}\right)|}$$

(6) $$\mathrm{RMSE}=\sqrt{\frac{1}{n}\sum {\left(\text{Predicted}-\text{Observed}\right)}^{2}}$$

Additionally, performance of VT‐PBPK models was evaluated based on the percent of individuals for whom PK parameters were predicted within the 1.25‐fold and more commonly used twofold error. Bland–Altman plots were provided to demonstrate the residuals against the mean of predicted and observed PK parameters.

### Ethics statement

The study complied with the Declaration of Helsinki and was approved by the Regional Committee for Medical and Health Research Ethics (2013/2379/REK). All patients signed a written informed consent before the study.

## RESULTS

### Database of 4β‐hydroxycholesterol/cholesterol levels

**Figure 2:**
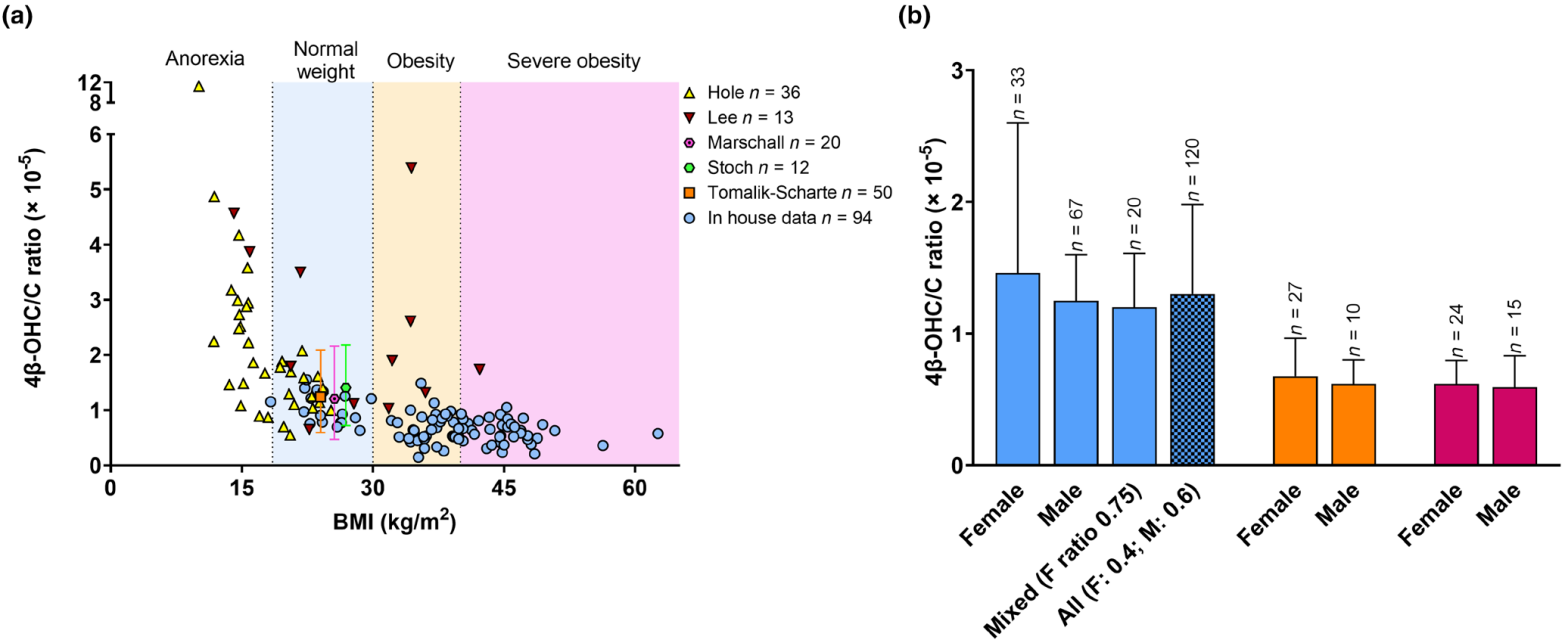
Relationship between body mass index (BMI) and 4β‐hydroxycholesterol/cholesterol ratio (4β‐OHC/C) data: (**a**) summary of literature^20^, ^21^, ^38^, ^39^, ^40^ (data digitized if reported individually) and COCKTAIL study^25^ participants, including the diet, surgery, and control groups. (**b**) Sex differences in 4β‐hydroxycholesterol/cholesterol ratio levels. Bars are colored by obesity category: non‐obese (blue), obese (orange), and severely obese (magenta).

Literature 4β‐OHC data were initially collated from 1,780 individuals. Of these, 1,496 were excluded because BMI ranges were not reported or data from participants with/without obesity were combined.^12^, ^19^, ^33^, ^34^ An additional 153 individuals were excluded due to missing total cholesterol measurements.^35^, ^36^, ^37^ After applying these criteria, literature data from 131 individuals^20^, ^21^, ^38^, ^39^, ^40^ were combined with the COCKTAIL dataset, resulting in 225 individuals with complete 4β‐OHC/C data, with a BMI ranging from 10 to 62.6 kg/m^2^. A decrease in biomarker levels with increasing BMI was observed, while levels remained similar between groups with obesity/severe obesity (**Figure** **2** **a**). Although biomarker levels were generally higher in female (1.2‐fold), the difference was not statistically significant (*P* = 0.17; **Figure** **2** **b**). Therefore, the weighted mean of 4β‐OHC/C in all individuals without obesity (1.3 × 10^−5^, 52% CV, *n* = 120) served as the healthy reference value. The corresponding unnormalized 4β‐OHC concentrations are provided in **Figure** **S1**.

### Hepatic CYP3A4 abundance in obesity and comparison to its activity markers

Hepatic CYP3A4 abundance measured in tissue samples pre‐surgery from all subjects in groups with obesity/severe obesity (*n* = 54) had a positive relationship with 4β‐OHC/C (*r*_s_ = 0.45, *P* < 0.001, **Figure** **S2**). A similar trend was observed between hepatic CYP3A4 abundance and weight‐normalized systemic clearance of midazolam, although the latter correlation was not statistically significant (*P* = 0.19).

**Figure 3:**
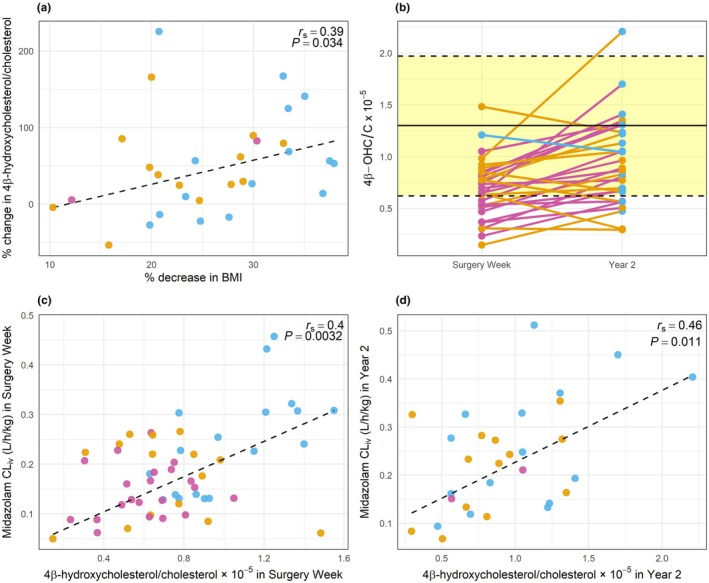
Comparison of (**a**) the changes in body mass index (BMI) and endogenous biomarker levels 2 years after the Roux‐en‐Y gastric bypass (RYGB) surgery (*n* = 30), (**b**) biomarker levels pre‐ and post‐surgery, relationship between 4β‐hydroxycholesterol/cholesterol ratio, and weight‐normalized intravenous clearance (CL_iv_) of midazolam (**c**) pre‐surgery and (**d**) 2 years post‐surgery. Each point represents an individual participant, colored by obesity category: non‐obese (blue), obese (orange), and severely obese (magenta). The black dashed line in panels **a**, **c**, and **d** represents the median trend line (0.5 quantile) obtained via quantile regression, providing an estimate of monotonic association. Spearman correlation coefficients (*r*_s_) and associated *P*‐values are shown in these panels. The yellow area in panel **b** represents the mean ± standard deviation of biomarker levels (dashed black lines) reported in healthy to overweight subjects from the database analysis.

All patients who underwent RYGB surgery experienced weight loss 2 years post‐surgery, with up to a 40% decrease in BMI. 4β‐OHC/C levels increased over time post‐surgery in 80% of individuals, although the degree varied (**Figure** **3** **a**). The number of individuals with 4β‐OHC/C levels within or above the healthy reference range (weighted mean ± SD) increased to 23/30 in year 2 (**Figure** **3** **b**). Weight‐normalized midazolam clearance values increased over time in 57% of individuals, reflecting biomarker trends (**Figure** **S3**), whereas the extent of change in remaining individuals showed no consistent relationship with 4β‐OHC/C. However, moderate (*r*_s_ = 0.4) and statistically significant correlations (*P* < 0.05) were noted between 4β‐OHC/C and weight‐normalized midazolam clearance at both surgery week (*n* = 54) and in year 2 (*n* = 30) (**Figure** **3** **c,d**).

### Validation of endogenous biomarker data for CYP3A4 phenotyping within VT‐PBPK models

**Figure 4:**
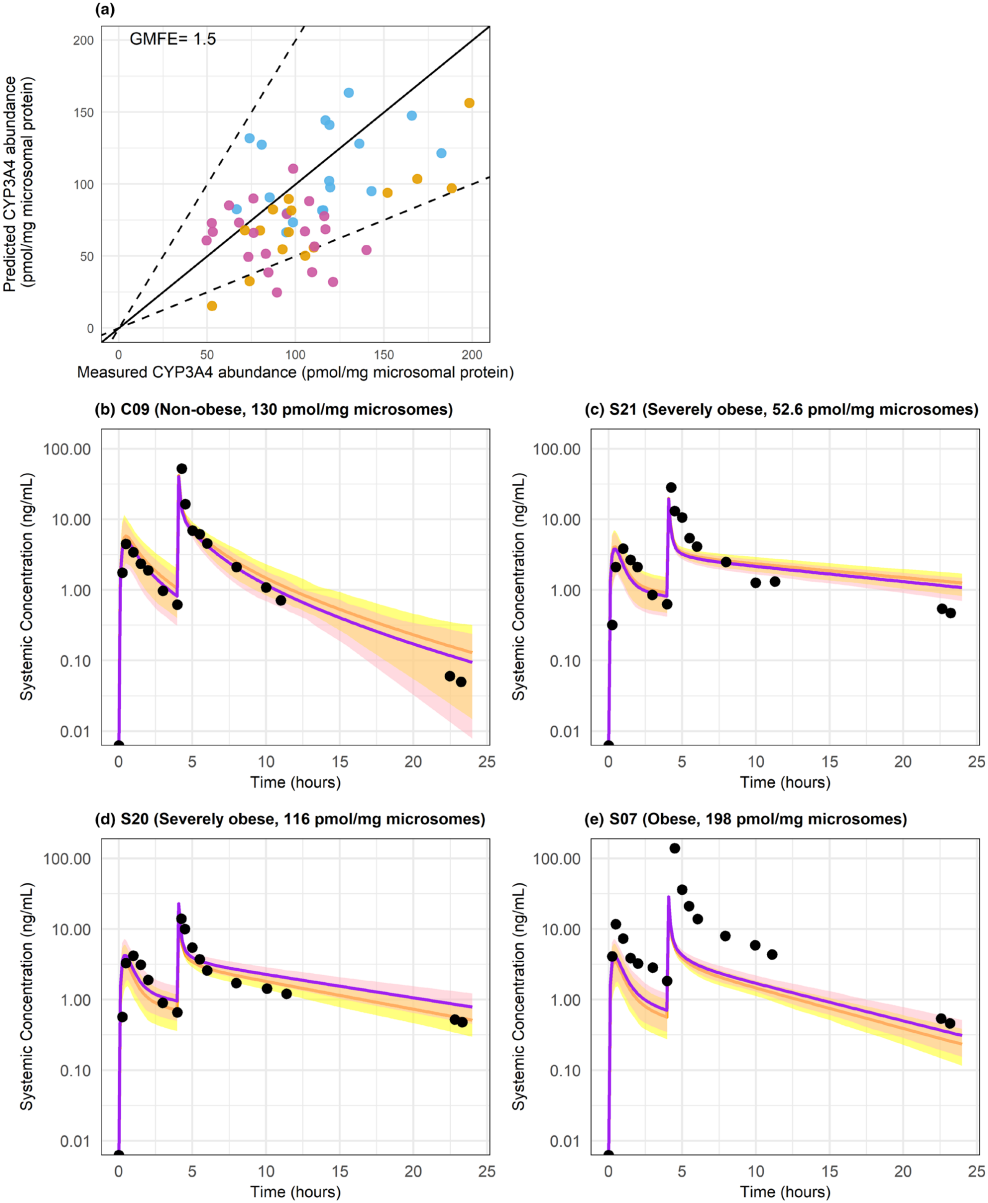
Evaluation of CYP3A4 abundance data informed by 4β‐hydroxycholesterol/cholesterol ratio. Relationship between (**a**) biomarker‐predicted and measured hepatic CYP3A4 abundance in the liver. Each point represents an individual donor, color‐coded by their obesity status: non‐obese (blue), obese (orange), and severely obese (magenta). The dashed lines indicate the twofold from the solid black line of unity. GMFE denotes for the geometric mean fold error between predicted and measured values (*n* = 54). (**b–e**) illustrate representative individuals showing the range of measured CYP3A4 protein abundance as indicated within brackets. Their predicted vs. observed plasma concentration time profiles of midazolam using the virtual twin PBPK models with either measured (orange line) or predicted (purple line) CYP3A4 abundance via biomarker approach. Areas representing the 5^th^ and 95^th^ percentiles of predictions are shaded with matching colors to predicted lines. Full circles represent observed data from the COCKTAIL trial.^25^

Individual 4β‐OHC/C data were used in VT‐PBPK models to predict the individual CYP3A4 abundance (Eq. 3) and compared against the actual protein measurements pre‐surgery. Good agreement was noted between measured and biomarker‐predicted CYP3A4 abundance (GMFE = 1.5), with < 15% of values predicted outside of twofold of the corresponding measurements (**Figure** **4** **a**). Together with other subject‐specific data (**Table** **S4**), VTs were constructed to simulate midazolam PK, following the validation of the midazolam model across patient populations (**Figure** **S4**).

Using biomarker‐informed hepatic CYP3A4 and other subject specific data (**Table** **S4**), the VT‐PBPK models predicted most of the observed *in vivo* midazolam concentrations within the 5th–95th percentile interval for 69% of individuals at the surgery week, comparable to CYP3A4 proteomics‐informed VT models (72%). Predicted PK profiles generated using either of the approaches were generally consistent with each other over the range of CYP3A4 abundance, as shown in representative profiles in **Figure** **4** **b–e**. Simulated profiles for all individuals using both proteomics and biomarker‐predicted CYP3A4 as inputs in VT‐PBPK models are in **Figures** **S5–S7**.

**Figure 5:**
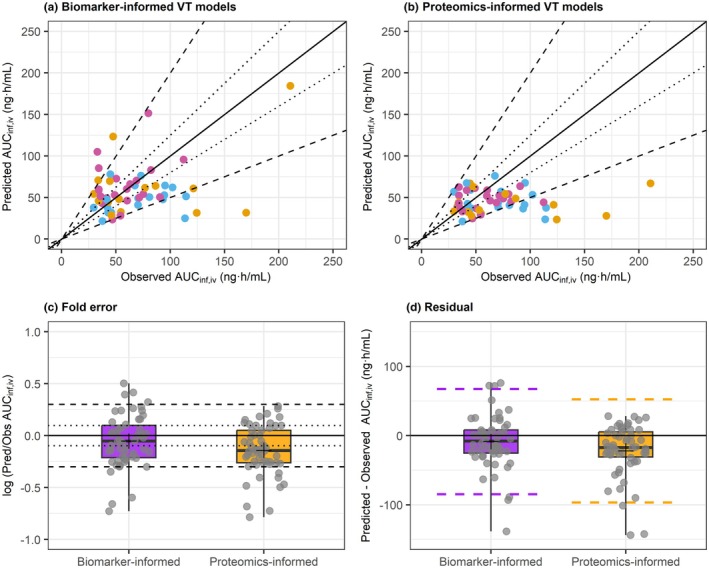
Comparison of predicted and observed AUC_inf,iv_ of midazolam pre RYGB surgery using (**a**) biomarker‐informed VT‐PBPK models or (**b**) proteomics‐informed VT PBPK models with the distribution of (**c**) fold errors and (**d**) residuals across all study individuals (*n* = 54). In (**a–c**) dashed and dotted lines denote twofold and 1.25‐fold difference around the solid line of unity, respectively. In (**a, b**) symbol colors represent obesity status in surgery week as non‐obese (blue), obese (orange), and severely obese (magenta). In (**c, d**) within each box, horizontal black lines denote median values and + symbol denotes mean values; boxes extend from the 25^th^ to the 75th percentile of each group's distribution of values, vertical extending lines denote the minimum and maximum values. In (**d**) the dashed lines represent ±1.96 standard deviation around the mean of residuals.

Biomarker‐informed VT models predicted midazolam *in vivo* AUC_inf,iv_ within twofold of the observed value in 46/54 individuals (GMFE = 1.58); 83% of those were individuals with obesity/severe obesity (GMFE = 1.59, *n* = 36). Only 30% of individual predictions were within 1.25‐fold of the observed data. VT models based on measured CYP3A4 abundance showed comparable performance, with 86% of individuals with obesity/severe obesity predicted within twofold (GMFE = 1.62, *n* = 36) and 83% across all individuals, with 20% predicted within 1.25‐fold (**Table** **1**, **Figure** **5**). Similar trends were noted for predictive performance of biomarker‐ and proteomics‐informed models for the *C*_max_ (78% within twofold) and AUC_inf,oral_ (50–59% within twofold) after oral dose (**Table** **1**).

**Table 1:** Summary performance of VT‐PBPK simulations in the same individuals with available data pre‐ and 2‐year post Roux‐en‐Y gastric bypass surgery reflecting the changes in obesity status^a^

Pre‐surgery	Biomarker‐informed VT‐PBPK models					Proteomics‐informed VT‐PBPK models				
	% within 1.25‐fold	% within twofold	AFE	GMFE	RMSE	% within 1.25‐fold	% within twofold	AFE	GMFE	RMSE
Non‐obese (*n* = 18)										
*C*_max,oral_	33	78	1.35	1.53	3.38	22	89	1.32	1.52	3.06
AUC_inf,oral_	17	61	1.74	1.89	9.81	33	50	1.67	1.78	8.56
AUC_inf,iv_	22	89	0.71	1.57	35.1	22	78	0.68	1.74	40.0
Obese (*n* = 15)										
*C*_max,oral_	40	80	0.77	1.49	2.90	27	73	0.63	1.69	3.54
AUC_inf,oral_	13	53	1.31	1.81	11.8	33	67	0.92	1.60	7.20
AUC_inf,iv_	27	73	0.85	1.77	53.0	13	73	0.62	1.90	64.1
Severely obese (*n* = 21)										
*C*_max,oral_	33	76	0.94	1.58	2.97	33	71	0.82	1.58	2.91
AUC_inf,oral_	14	38	1.94	2.35	11.1	24	62	1.48	1.75	8.17
AUC_inf,iv_	38	90	1.10	1.47	30.3	24	95	0.84	1.44	24.3
All individuals (*n* = 54)										
*C*_max,oral_	35	78	1.00	1.54	3.10	28	78	0.89	1.59	3.15
AUC_inf,oral_	15	50	1.68	2.03	12.6	30	59	1.35	1.71	8.05
AUC_inf,iv_	30	85	0.89	1.58	39.4	20	83	0.72	1.66	43.7

Two years post‐surgery	Biomarker‐informed VT‐PBPK models assuming no recovery of intestinal CYP3A4 post‐surgery					Biomarker‐informed VT‐PBPK models with intestinal CYP3A4/5 recovery post‐surgery				
Non‐obese (*n* = 15)										
*C*_max,oral_	27	53	1.81	1.91	6.07	20	67	1.61	1.76	4.99
AUC_inf,oral_	13	67	1.49	1.81	12.2	13	73	1.30	1.78	10.0
AUC_inf,iv_	27	87	0.73	1.62	32.4	27	87	0.72	1.62	37.3
Obese and severely obese (*n* = 15)										
*C*_max,oral_	7	67	1.68	1.83	5.40	20	73	1.50	1.68	4.41
AUC_inf,oral_	7	47	2.14	2.14	20.0	13	67	1.87	1.87	16.3
AUC_inf,iv_	53	87	1.02	1.36	31.5	53	87	1.01	1.35	31.2
All individuals (*n* = 30)										
*C*_max,oral_	17	60	1.74	1.87	5.75	20	70	1.55	1.72	4.71
AUC_inf,oral_	10	57	1.78	1.97	16.6	13	70	1.56	1.82	13.6
AUC_inf,iv_	40	87	0.86	1.48	34.4	40	87	0.85	1.48	34.4

AFE, average fold error; GMFE, geometric mean fold error; RMSE, root mean squared error.

^a^ Individual classified with obesity when body mass index (BMI) is 30.0–39.9 kg/m^2^ and with severe obesity when BMI is ≥ 40.0 kg/m^2^.

### Application of endogenous biomarker for CYP3A4 phenotyping in year 2 in the absence of tissue biopsy

**Figure 6:**
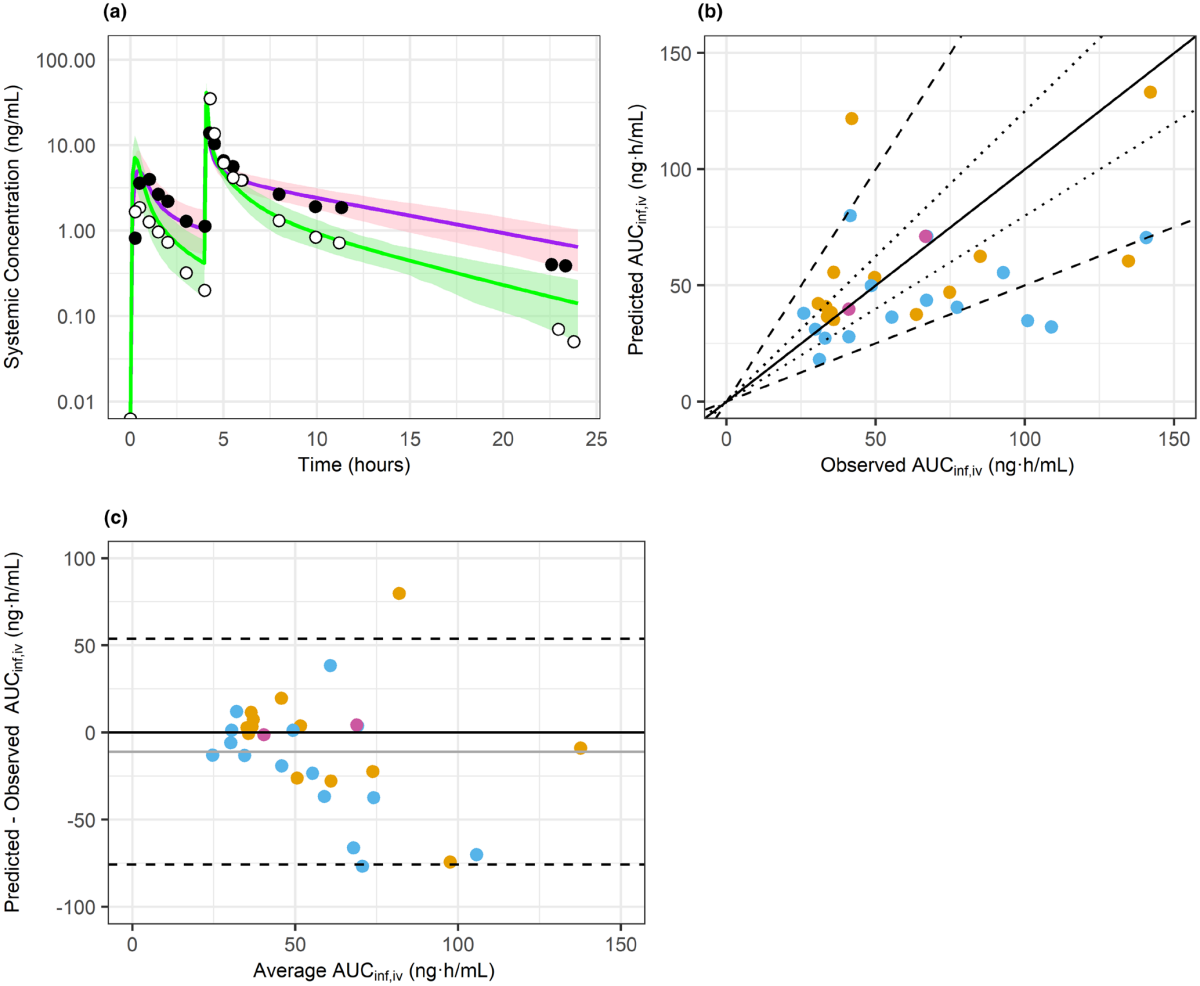
Biomarker‐informed VT‐PBPK model performances in 2 years post‐Roux‐en‐Y gastric bypass surgery. (**a**) A simulation of midazolam pharmacokinetics in a representative individual. The virtual twin PBPK models used either predicted hepatic CYP3A4 abundance based on 4β‐hydroxycholesterol/cholesterol ratio data at the surgery week (purple) and in year 2 (green). Simulations in year 2 (oral component) were based on the assumption of full recovery of intestinal CYP3A4/5 2 years post‐surgery. (**b**) Comparison of predicted and observed AUC_inf,iv_ of midazolam post‐surgery across all individuals (*n* = 30) and (**c**) Bland–Altman plot illustrating residuals against the average of predicted and observed AUC_inf,iv_ of midazolam. In (**a**) solid lines represent the predicted profiles. Black and white circles represent observed midazolam data at the surgery week and in year 2 in a representative individual, respectively. Areas representing the 5th and 95th percentiles of predictions are shaded with matching colors to predicted lines. In (**b**) black solid, dotted, and dashed lines denote the line of unity, 1.25‐fold, and twofold difference, respectively. In (**c**) the dashed lines represent ±1.96 standard deviation around the mean of residuals, represented by gray solid line.

4β‐OHC/C increased in most individuals from time of surgery to year 2, suggesting an increase in hepatic CYP3A activity over time post‐surgery (**Figure** **3** **b**). Following successful verification of the biomarker approach in surgery week, VT‐PBPK models, updated with changed physiological parameters and hepatic CYP3A4 levels post‐surgery, successfully predicted the altered midazolam PK in the same individuals 2 years after RYGB surgery, as illustrated by a representative profile in **Figure** **6** **a**. Profiles for all individuals are provided in **Figures** **S8** and **S9**. Biomarker‐informed VT‐PBPK models predicted midazolam exposure after iv dosing (AUC_inf,iv_) within twofold of the observed for 87% and within mean ± 1.96 SD of difference in 93% of individuals (**Figure** **6** **b,c**, **Table** **1**). However, observed midazolam PK parameters after oral administration were predicted within twofold for ~60% of the individuals. This performance improved to 70% once the VT‐PBPK RYGB models assumed full recovery of intestinal CYP3A4/5 2 years post‐surgery (**Table** **1**, **Figure** **S10**). For 83% of individuals, the majority of the observed plasma concentration data were within the 5th–95th percentile of the simulations using the RYGB surgery model with intestinal CYP3A4/5 recovery.

The consideration of RYGB surgery in VT‐PBPK models mainly impacted the predicted fraction escaping intestinal metabolism (Fg) of midazolam (~0.59 vs. pre‐surgery level of ~0.44). The overall predicted fraction absorbed (fa) remained to be 1. In pre‐surgery, the duodenum contributed ~41% of the fraction absorbed, whereas in post‐surgery, the duodenum had a minimal role. Similarly, midazolam was not metabolized in the duodenum post‐surgery compared to ~24% contribution pre‐surgery. Under the assumption of complete recovery of total intestinal CYP3A4/5 abundance 2 years post‐surgery, the overall fa and Fg were predicted as 1 and ~ 0.51, with the main contributing regions being jejunum I (~48% absorbed, ~15% metabolized) and jejunum II (~36% absorbed, ~24% metabolized), respectively.

## DISCUSSION

### Evaluation of 4β‐hydroxy cholesterol/cholesterol as an endogenous hepatic CYP3A4 biomarker in obesity

The current study focused on monitoring 4β‐OHC/C to assess hepatic CYP3A4 activity over time in patients with changing obesity status. Participants reported stable body weight (< 5 kg change) over 3 months before enrollment,^25^ which is longer than the half‐life of 4β‐OHC.^14^ Therefore, their plasma 4β‐OHC/C levels were considered reflective of steady‐state CYP3A4 activity. Database analysis revealed a progressive decrease in 4β‐OHC/C with increasing BMI, with a plateau with BMI ≥ 30 (**Figure** **1**). Biomarker levels (both normalized for cholesterol and unnormalized) were approximately twofold lower in individuals with obesity/severe obesity than in individuals without obesity (**Figure** **2**), suggesting comparable suppression of CYP3A4 activity irrespective of obesity severity. Suppression of CYP3A4 activity in obesity was previously rationalized by the inflammation status, illustrated by a weak inverse correlation between 4β‐OHC concentrations and the inflammation marker high sensitivity C‐reactive protein levels.^17^

In this study, 4β‐OHC/C was used to phenotype hepatic CYP3A4 due to kinetic rationale and because it correlated more strongly with hepatic CYP3A4 abundance than unnormalized 4β‐OHC. To obtain a population‐independent measure of CYP3A activity, changes in the 4β‐OHC/C relative to the healthy reference (Eq. 3) were used to inform the VT‐PBPK models, consistent with phenotyping approaches based on exogenous CYP3A substrates (e.g., the 1′‐hydroxymidazolam/midazolam ratio).^1^ This approach was considered more robust than relying on a regression line between CYP3A4 expression and biomarker levels in 54 individuals included in the current dataset.

Midazolam, a widely used exogenous CYP3A4 substrate,^10^ is a medium‐to‐high hepatic extraction ratio drug. Therefore, its systemic clearance is determined not only by intrinsic metabolic capacity but also by hepatic blood flow and plasma protein binding.^41^ In contrast, formation of 4β‐OHC is not influenced by hemodynamic factors and may therefore provide a more direct reflection of changes in hepatic CYP3A4 in obesity.^17^ Consistent with this, no significant correlation was observed between hepatic CYP3A4 abundance and weight‐normalized midazolam clearance before surgery (*P* = 0.19; *n* = 54, data not shown), whereas the corresponding correlation between the 4β‐OHC/C and midazolam clearance was significant (**Figure** **3** **c**). These findings indicate that the 4β‐OHC/C may capture long‐term functional CYP3A activity in obesity, which may not be adequately reflected by hepatic CYP3A4 abundance measured from a single biopsy providing only a static snapshot.

### Performance of biomarker‐informed VT PBPK models for individuals with obesity

Biomarker‐informed VT‐PBPK models slightly outperformed proteomics‐informed models in prediction of midazolam systemic exposure after iv dosing, especially for individuals demonstrating low midazolam systemic clearance (**Figure** **5**), as the proteomics‐informed models did not capture inter‐individual variability in observed AUC_inf,iv_ data. Although > 80% of individual predictions were within twofold with both approaches, only 30% or less fell within 1.25‐fold. These model performances were consistent across obesity categories (**Table** **1**), indicating that uncertainty in exposure predictions is driven by other drug‐ and system‐specific PBPK parameters for which we did not have measurements (e.g., individual hepatic blood flow, intestinal CYP3A4 abundance).

The ability of the biomarker and proteomics‐informed VT‐PBPK models to predict oral *C*_max_ and AUC_inf,oral_ were similar (**Table** **1**). Considering that midazolam exhibits almost complete absorption, this performance could be attributed to Fg and the intestinal CYP3A4/5 abundance used in VT‐PBPK models. 4β‐OHC/C is widely considered a marker of hepatic rather than intestinal CYP3A4 activity,^42^, ^43^ supported also by the fact that only 20% of total body cholesterol comes from dietary intake.^43^ Although some intestinal contribution cannot be ruled out, its overall role in 4β‐OHC formation appears negligible.^17^ Therefore, biomarker levels were used solely to individualize hepatic CYP3A4, whereas population levels were used for intestinal CYP3A4 abundance in the absence of individual data.

We have previously evaluated the impact of individualization of VT‐PBPK models with subject‐specific data (serum albumin, creatinine, CYP3A4/5 hepatic abundance) and their ability to predict midazolam and digoxin PK including the current study individuals.^2^ In that study, consideration of subject specific data resulted in marginal improvement in overall predictive performance. However, VT models provided more robust predictions with tighter confidence intervals and a few outliers with very low CYP3A4 expression benefitted from VT‐PBPK modeling informed with individual proteomics data.^2^ Considering the invasive nature of tissue biopsies, generation of individual abundance is challenging. In contrast, endogenous biomarkers provide a non‐invasive, plasma‐based measure of functional metabolic activity, making this approach more suitable for real‐world applications. The current study illustrated that monitoring of changes in 4β‐OHC/C relative to healthy reference value is a valuable surrogate to inform the PBPK models, as this approach resulted in comparable performance to proteomics‐informed models across individuals with/without obesity (**Figure** **4**).

### Dynamic changes in biomarker levels for continuous monitoring of hepatic CYP3A4 activity

Biomarker‐informed VT‐PBPK modeling allowed continuous monitoring of individual hepatic CYP3A4 activity and captured subject‐specific alterations 2 years post‐RYGB surgery (**Figure** **3** **a,b**). All individuals experienced weight loss compared with pre‐surgery, although some remained in the obese or severely obese category. In most individuals (*n* = 17), both biomarker and weight‐normalized systemic clearance of midazolam were increased, suggesting a recovery of CYP3A4 activity with reduced BMI. Notably, in 13/30 individuals, either the biomarker (*n* = 5), the weight‐normalized systemic clearance of midazolam (*n* = 7), or both (*n* = 1) were reduced, highlighting interindividual variability in the response of CYP3A4 activity to weight loss (**Figure** **S2**). Together with changes in other physiological parameters associated with altered obesity status, the VT‐PBPK models successfully predicted midazolam AUC_inf,iv_ within 1.25‐ and twofold of observed in 40% and 87%, respectively (**Table** **1**). These results demonstrated the utility of a biomarker‐informed approach in capturing subject‐specific recovery of hepatic CYP3A4 activity due to weight loss and reduced inflammation, consistent with the literature observations 1–6 months post bariatric surgery.^44^, ^45^

Surgery‐related changes in the gut physiology were only critical for predicting PK of oral midazolam. In the current study, RYGB surgery involved the bypass of ~86 cm of the proximal small intestine. Therefore, the function of the CYP3A enzymes predominantly expressed in this area was initially considered diminished while the absolute enzyme abundance in the remaining regions was unaltered, leading to a 1.3‐fold increase in predicted Fg of midazolam compared to pre‐surgery, while overall predicted fa remained unchanged. However, midazolam exposure after oral administration was over‐predicted in 40% of individuals, as illustrated for both *C*_max_ and AUC_inf,oral_ (**Table** **1**, **Figure** **S9**).

Longitudinal studies measuring intestinal and hepatic CYP3A4 before and after RYGB showed an apparent increase in CYP3A4 activity at 5–8 weeks post‐surgery, followed by a return to baseline phenotypic values by ~25–30 weeks.^46^ These transient changes were reported based on oral midazolam, which may also reflect the recovery of hepatic CYP3A4 considering that there is no direct marker for recovery of intestinal CYP3A4 expression/function.^47^ In another study, midazolam *C*_max_ increased by 66% and 71% at 3‐ and 12‐month post‐RYGB compared to pre‐surgery,^48^ suggesting no difference in CYP3A recovery within a 9‐month period. In the current study, informing the VT‐PBPK models solely with subject‐specific post‐surgery recovery of hepatic CYP3A4 was not sufficient to explain the magnitude of change in oral midazolam kinetics 2 years post‐surgery, despite well‐predicted iv clearance of midazolam. Thus, a hypothetical recovery of intestinal CYP3A4/5 abundances 2 years post‐surgery was simulated. Once the total intestinal abundance of CYP3A4/5 was reset to pre‐surgery levels, absolute abundances in the remaining regions (distal jejunum and ileum) increased by 1.45‐fold, resulting in minimal change in fraction metabolized in these regions. Even so, the prediction of oral midazolam PK was improved, with 70% *C*_max_ and AUC_inf,oral_ predicted within twofold of the observed (**Figure** **S9**). It should be noted that the current RYGB model assumed no change in the intestinal blood flow and regional permeability, which may also be impacted by the surgery.

The current prospective bottom‐up VT‐PBPK models predicted well midazolam PK even though no subject‐specific measurements of the intestinal parameters were available. Since the main intestinal regions contributing to midazolam fa and Fg shifted due to RYGB surgery, pre‐systemic local drug concentrations may also be altered. Consequently, the magnitude of DDIs after surgery may change and will depend also on the physicochemical and biopharmaceutical properties of precipitant drugs. Current biomarker‐informed VT‐PBPK models can be used to simulate the PK and associated DDIs for CYP3A4 substrates in further complex scenarios both in pre‐ and post‐surgery conditions, which are challenging to study clinically.

## CONCLUSIONS

This study demonstrated, for the first time, the utility of endogenous biomarker 4β‐OHC/C as a non‐invasive approach for continuous CYP3A4 phenotyping. Combined with VT‐PBPK modeling, it enabled subject‐specific longitudinal characterization of CYP3A4 activity and supports the development of precision dosing strategies for individuals with obesity. Upon the availability of clinical data, this biomarker‐PBPK approach can be translated to other patient populations where the expression of CYP3A4 changes relative to a healthy physiological state.

## FUNDING

This work was supported by the Research Council of Norway under Grant 315792.

## CONFLICTS OF INTEREST

R.J.‐L. is an employee of AstraZeneca. A.R.‐H. is an employee at Certara Predictive Technologies (CPT), Simcyp Division. All other authors declared no competing interests for this work.

## AUTHOR CONTRIBUTIONS

N.I., H.K. R.J.‐L., J.K.H., A.R.‐H., I.R., and A.G. wrote the manuscript; N.I., H.K., I.R., and A.G. designed the research; N.I. and H.K. performed the research; N.I., H.K., and A.G. analyzed the data.

## Supporting information


Data S1.

## Data Availability

Access to data collected from this study, including anonymized individual participant data, may potentially be made available following publication on e‐mail request to the corresponding author. After approval of a proposal, data will be shared with investigators whose proposed use of the data has been approved by the COCKTAIL steering committee, according to the consent given by the participants and Norwegian laws and legislations.
