# Supplementary material for: Application of Virtual Twin PBPK Models in Individuals with Obesity via CYP3A4 Phenotyping Using Endogenous Biomarker Data
Source: Clin Pharmacol Ther. 2026 Jun 18;120(3):762–73. doi: 10.1002/cpt.70369 (PMC13339604; doi:10.1002/cpt.70369)
Supplement: Supplementary file 1 — Data S1. [file CPT-120-762-s001.pdf]

**SUPPLEMENTARY MATERIALS**

# Application of virtual twin PBPK models in individuals with obesity via CYP3A4 phenotyping using endogenous biomarker data

Nihan Izat[1], Haribhau Kangne[1], Rasmus Jansson-Löfmark[2], Jens K Hertel[3], Amin Rostami-Hodjegan[1,4], Ida Robertsen[5] and Aleksandra Galetin[1]

[1]Centre for Applied Pharmacokinetic Research, The University of Manchester, Manchester, United Kingdom

[2]Drug Metabolism and Pharmacokinetics, Research and Early Development, Cardiovascular, Renal and Metabolism (CVRM), BioPharmaceuticals R&D, AstraZeneca, Gothenburg, Sweden

[3]Department of Endocrinology, Obesity and Nutrition, Vestfold Hospital Trust, Tønsberg, Norway

[4]Certara Predictive Technologies, Sheffield, United Kingdom

[5]Section for Pharmacology and Pharmaceutical Biosciences, Department of Pharmacy, University of Oslo, Oslo, Norway

**CONTENT**

PAGES


| | |
|---|---|
| Supplementary methods | 4 |
| Table S1. Post-surgery (Roux-en-Y gastric bypass) system parameters used in physiologically based pharmacokinetic model simulations | 5 |
| Table S2. Input parameters of midazolam used in physiologically based pharmacokinetic model simulations | 7 |
| Table S3. *In vivo* pharmacokinetic studies after oral midazolam administration represented in physiologically-based pharmacokinetic (PBPK) model trial design | 8 |
| Table S4. Summary of VT-PBPK model input parameters for individuals with obesity and severe obesity | 9 |
| Figure S1. Sex differences in 4β-hydroxycholesterol (ng/mL) levels | 10 |
| Figure S2. Relationship between hepatic CYP3A4 abundance and 4β-hydroxycholesterol/cholesterol ratio. | 11 |
| Figure S3. Relationship between the percent change in 4β-hydroxycholesterol/cholesterol ratio, and weight-normalised intravenous clearance (CLiv) of midazolam at 2-years post-surgery compared to pre-surgery | 12 |
| Figure S4. Validation of full-body PBPK with ADAM model for oral midazolam | 13 |
| Figure S5. Simulation of the midazolam pharmacokinetics of non-obese individuals using the virtual twin PBPK models using measured or predicted CYP3A4 abundance via biomarker data at the surgery week | 14 |
| Figure S6. Simulation of the midazolam pharmacokinetics in individuals with obesity using the virtual twin PBPK models using measured or predicted CYP3A4 abundance via biomarker data at the surgery week | 15 |

Figure S7. Simulation of the midazolam pharmacokinetics in individuals with severe obesity using the virtual twin PBPK models using measured or predicted CYP3A4 abundance via biomarker data at the surgery week 16

Figure S8. Simulation of the midazolam pharmacokinetics using the virtual twin PBPK models using predicted CYP3A4 abundance via biomarker data at the surgery week and in year 2 17

Figure S9. Simulation of the midazolam pharmacokinetics using the virtual twin PBPK models using predicted CYP3A4 abundance via biomarker data at the surgery week and in year 2 with the assumption of full recovery of intestinal CYP3A4/5 18

Figure S10. Comparison of predicted and observed $C_{max}$ and $AUC_{inf,oral}$ of oral midazolam 2 years post Roux-Y gastric bypass surgery assuming reduced or recovered intestinal CYP3A4/5 activity compared to pre-surgery 19

Supplementary references 20

**Supplementary methods**

A PK investigation was conducted following semi-simultaneous oral (1.5 mg) and intravenous (iv) dosing (1 mg at 4 h) of midazolam on the day before surgeries in groups with/without obesity and two years post-surgery in the group with obesity.[1] The other five drugs given in the cocktail dosing were not considered in the current PBPK models. Blood samples were collected from all patients for determination of plasma 4β-OHC, total cholesterol (0 h), and midazolam concentrations over 24 hours.

Standard clinical chemistry analyses, including total cholesterol,[2] serum albumin and serum creatinine were performed in fresh blood samples at the Department of Laboratory Medicine, Vestfold Hospital Trust, Tønsberg, Norway. 4β-OHC concentrations were normalised with total cholesterol as the molar ratio at surgery and year 2. The details of the study design,[1] quantification of 4β-OHC, cholesterol[2], and midazolam[3], CYP3A5 genotype[2] and proteomics assays[4] on liver tissue biopsies collected during surgeries have been published previously. In the proteomics assay, the absolute abundance of hepatic CYP3A4 was quantified in the liver homogenate fraction.[4] Therefore, expression levels were converted to units of pmol per mg of microsomal protein by a factor of 4.4[5] as applied previously.[6]

**Table S1.** Post-surgery (Roux-en-Y gastric bypass) system parameters used in physiologically based pharmacokinetic model simulations (Simcyp v23-2)

| Parameter[a] | Value (CV) | Comment |
|---|---|---|
| **Mean Residence Time** | | |
| Fasted mean gastric residence time (h) | 0.1167 (45%)[7] | |
| Small intestine Weibull distribution parameters | α: 2.92; β: 3.37 (39.93%) | Assuming reduced transit time (3h) based on the time needed to pass remaining intestinal regions without considering any change in intestinal motility compared to pre-surgery |
| **Anatomy of the Intestine[b]** | | |
| Total length of the intestine | Male: 4.458 (20.45%)<br>Female: 4.306 (19.33%) | Based on complete by-pass of duodenum (0.27 m) and partial by-pass of proximal jejunum (0.6 m) |
| Fraction of total small intestine length | Duodenum: 0.001<br>Jejunum: 0.282<br>Ileum: 0.718 | Duodenum length was set near zero due to Simulator's limit. Additionally, $P_{eff,\ duodenum}$ (10-4 cm/s) in compound file was set to 0.0001 |
| **CYP Phenotype** | | |
| CYP3A4 abundance (nmol/small intestine) | 45 (52%)<br>After recovery: 65.4 (52%) | Based on the loss of active enzyme abundance proportional to the by-passed region |
| CYP3A5 abundance (nmol/small intestine) | 16 (32%)<br>After recovery: 23.3 (32%) | Post-surgery simulations were repeated with %100 recovery of enzyme abundances by re-setting them at pre-surgery levels |
| **ADAM Model Parameters** | | |
| **General** | | |
| CYP3A (Total %) | Duodenum: 0.001<br>Jejunum I: 14.249<br>Jejunum II: 39.59<br>Ileum I-IV: 11.54 | Absolute enzyme abundances in Jejunum II to Ileum IV remains unaltered compared to the pre-surgery model |
| Transit Time (Total %) | Duodenum: 0.001<br>Jejunum I: 7.418<br>Jejunum II: 20.905<br>Ileum I-IV: 17.919 | Absolute transit times in Jejunum II to Ileum IV remains unaltered compared to the pre-surgery model. The post-surgery length ratio between Jejunum II and Jejunum I (2.8-fold) was applied in transit time as the simulator only allows total Jejunum length parameterisation |

| | | |
|---|---|---|
| Blood Flow ($Q_{villi}$ %) | Duodenum: 10.518<br>Jejunum I: 35.944<br>Jejunum II: 24.786<br>Ileum I-II: 7.3165<br>Ileum III: 7.1978<br>Ileum IV: 6.921 | Assumed unaltered compared to the pre-surgery models |
| **Luminal pH** | | |
| pH Fasted, stomach | 6.4 (38%)[7] | |
| **Luminal Bile Salts** | | |
| [Bile] Fasted (nM) | Stomach: 0.002[7]<br>Duodenum: 0<br>Jejunum I-II: 0 | Alimentary limb in the study surgery group was 120 cm, thereby bile salt was removed in the corresponding intestinal region in the model |
| **Luminal Fluid Volume** | | |
| Basal Fluid Volumes (mL) | | |
| Stomach, Fasted | 9.9[7] | |
| **Trial Design** | | |
| Fluid intake with dose (mL) | 30 (30%)[7] | |

[a]Non-listed gastrointestinal system parameters remained unaltered compared to pre-surgery model

[b]The new method option for the length and diameter of small intestinal and colon (Simcyp v23) parameters was applied to both pre- and post-surgery models to maintain consistent assumptions regarding initial intestinal length for the same individual represented as a virtual twin. In the post-surgery model, only surgery-related anatomical modifications were implemented, while the initial intestinal length was preserved as the reference point

**Table S2.** Input parameters of midazolam used in physiologically based pharmacokinetic model simulations (Simcyp v23-2)

| | **Midazolam** |
|---|---|
| **MW (g/mol)** | 325.8 |
| **Compound type** | Monoprotic Base |
| **logP** | 3.53 |
| **$pKa_1$** | 6 |
| **$fu_p$** | 0.032 |
| **B/P** | 0.603 |
| **Plasma protein** | Human serum albumin |
| **Absorption model** | ADAM |
| **Caco-2 permeability** ($10^{-6}$ cm/s) | 213 |
| **Predicted $P_{eff}$** ($10^{-4}$ cm/s) | 6.045 |
| **Intrinsic solubility** (mg/mL) | 0.054 |
| **Formulation** | Oral solution |
| **Segregated Transit Time Model** | OFF |
| **$fu_{gut}$** | 1 |
| **Distribution model** | Full PBPK |
| **$V_{ss}$** (L/kg) | 1.1854 |
| **Kp scalar** | 0.25 |
| **Enzyme kinetics** | Recombinant systems data |
| **1'-OH pathway** | |
| **Vmax** (pmol/min/pmol CYP3A4) | 5.23 |
| **Km (μM)** | 2.16 |
| **1-OH pathway** | |
| **Vmax** (pmol/min/pmol CYP3A5) | 19.7 |
| **Km** (μM) | 4.16 |
| **4-OH pathway** | |
| **Vmax** (pmol/min/pmol CYP3A4) | 5.2 |
| **Km** (μM) | 31.8 |
| **4-OH pathway** | |
| **Vmax** (pmol/min/pmol CYP3A5) | 4.03 |
| **Km** (μM) | 38.4 |
| **Enzyme kinetics** | Human liver microsomes data |
| **Other pathway** | |
| **Vmax** (pmol/min/pmol UGT1A4) | 445 |
| **Km** (μM) | 40.3 |
| **$fu_{mic}$** | 1 |

*B/P, blood-to-plasma ratio; $CL_{Renal}$, observed renal clearance; $fu_{mic}$, incubational unbound fraction in microsomes or in recombinant systems; $fu_p$, unbound fraction in plasma; logP, the octanol–water partition coefficient; MW, molecular weight; pKa, the acid dissociation constant; $V_{ss}$, volume of distribution at steady state*

**Table S3.** *In vivo* pharmacokinetic studies after oral midazolam administration represented in physiologically-based pharmacokinetic (PBPK) model trial design

| Population | Dose (mg) | Number of subjects | Number of trials | Age range | Female fraction | Reference |
|---|---|---|---|---|---|---|
| Sim-Healthy | 3 | 62[a] | 4 | 18-34 | 0.44 | [8] |
| Sim-Healthy | 4 | 16 | 15 | 20-40 | 0.5 | [9] |
| Sim-Healthy | 5[b] | 12 | 20 | 19-42 | 0.5 | [10] |
| Sim-Healthy | 7.5 | 12 | 20 | 19-24 | 0.42 | [11] |
| Sim-Healthy | 10 | 6 | 20 | 21-22 | 0.17 | [12] |
| Sim-Healthy | 20 | 6 | 20 | 22-27 | 0 | [13] |
| Sim-Morbidly Obese | 10 | 1[c] | 240 | 36-36 | 1 | [14] |
| Sim-Morbidly Obese | 2 | 12 | 20 | 37-55 | 0.75 | [15] |
| Sim-Obese and RYGB | 2 | 10 | 20 | 37-55 | 0.8 | [15] |

[a]Based on CYP3A5*3/*3 genotype
[b]Followed with 2 mg iv (6h after the oral dose)
[c]Based on a representative individual profile

**Table S4.** Summary of VT-PBPK model input parameters for individuals with obesity and severe obesity. Data presented as mean (range).

| | Parameter | Non-obese | Obese | Severely obese |
|---|---|---|---|---|
| **Surgery week** | n | 18 | 15 | 21 |
| | Age (Years) | 43 (19-63) | 46 (32-63) | 45 (23-60) |
| | Sex, n (Male/Female) | 3/15 | 3/12 | 8/13 |
| | Ethnicity | White | White | White |
| | Body weight (kg) | 71 (47-97) | 105 (83-130) | 137 (101-166) |
| | BMI (kg/m²) | 24.7 (18.3-29.8) | 37 (32.8-39.8) | 46.2 (40.3-62.6) |
| | Serum creatinine (µmol/L) | 62 (37-91) | 57 (36-77) | 60 (42-81) |
| | Serum albumin (g/L) | 40 (35-45) | 39 (16-44) | 41 (35-44) |
| | Measured CYP3A4 (pmol/mg microsomal protein) | 114 (67-183) | 111 (53-198) | 90 (50-140) |
| | Predicted CYP3A4[a] (pmol/mg microsomal protein) | 109 (66-163) | 74 (15-156) | 64 (25-111) |
| | Lean liver volume (L) | 1.5 (1.1-1.9) | 1.8 (1.5-2.0) | 2 (1.5-2.4) |
| | CYP3A5 phenotype (% PM) | 89 | 87 | 91 |
| **Year 2** | n | 15 | 13 | 2 |
| | Age (Years) | 48 (34-65) | 48 (25-59) | 50 (49-50) |
| | Sex, n (Male/Female) | 3/12 | 7/6 | 0/2 |
| | Ethnicity | White | White | White |
| | Body weight (kg) | 77 (60-98) | 101 (73-122) | 108 (99-116) |
| | BMI (kg/m²) | 26.8 (23.5-29.6) | 33.5 (30-37.3) | 42.7 (41.9-43.6) |
| | Serum creatinine (µmol/L) | 61 (45-120) | 60 (46-73) | 65 (63-66) |
| | Serum albumin (g/L) | 36 (32-39) | 37 (33-42) | 34 (34-34) |
| | Predicted CYP3A4[a] (pmol/mg microsomal protein) | 113 (50-233) | 87 (31-142) | 85 (60-111) |
| | Lean liver volume (L) | 1.7 (1.4-1.9) | 1.8 (1.5-2.3) | 1.6 (1.5-1.8) |
| | CYP3A5 phenotype (% PM) | 87 | 92 | 100 |

n- Number of subjects; BMI-Body Mass Index; PM- Poor metabolizer

[a]Predicted using 4β-hydroxy cholesterol/cholesterol data

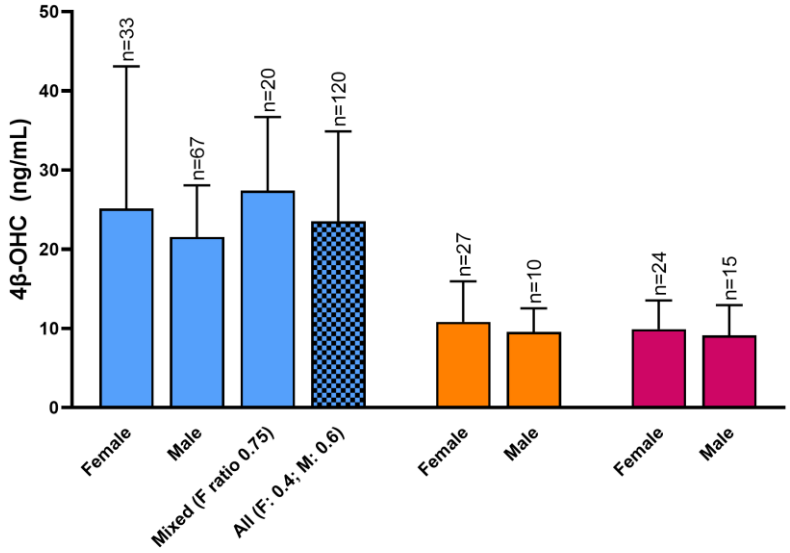


**Figure S1.** Sex differences in 4β-hydroxycholesterol (ng/mL) levels including summary of literature data (Hole et al. n=36[16]; Lee et al. n=13[17]; Marschall et al. n=20[18]; Stoch et al. n=12[19]; Tomalik-Scharte et al. n=50[20]) and Cocktail study participants, including the diet, surgery and control groups. Bars are coloured by obesity category: non-obese (blue), obese (orange), and severely obese (magenta).

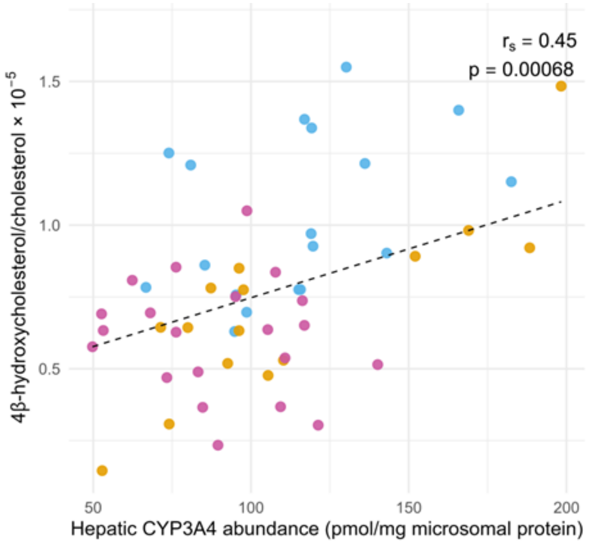


**Figure S2.** Relationship between hepatic CYP3A4 abundance and 4β-hydroxycholesterol/cholesterol ratio. All data are from the COCKTAIL trial.[1] Each point represents an individual participant, coloured by obesity category: non-obese (blue), obese (orange), and severely obese (magenta). The black dashed line in each panel represents the median trend line (0.5 quantile) obtained via quantile regression, providing an estimate of monotonic association. Spearman correlation coefficients (rs) and associated p-values are shown in each panel. No empirically derived regression from the illustrated data was used for CYP3A4 phenotyping. Please refer to Eq.3 in the main text for CYP3A4 phenotyping.

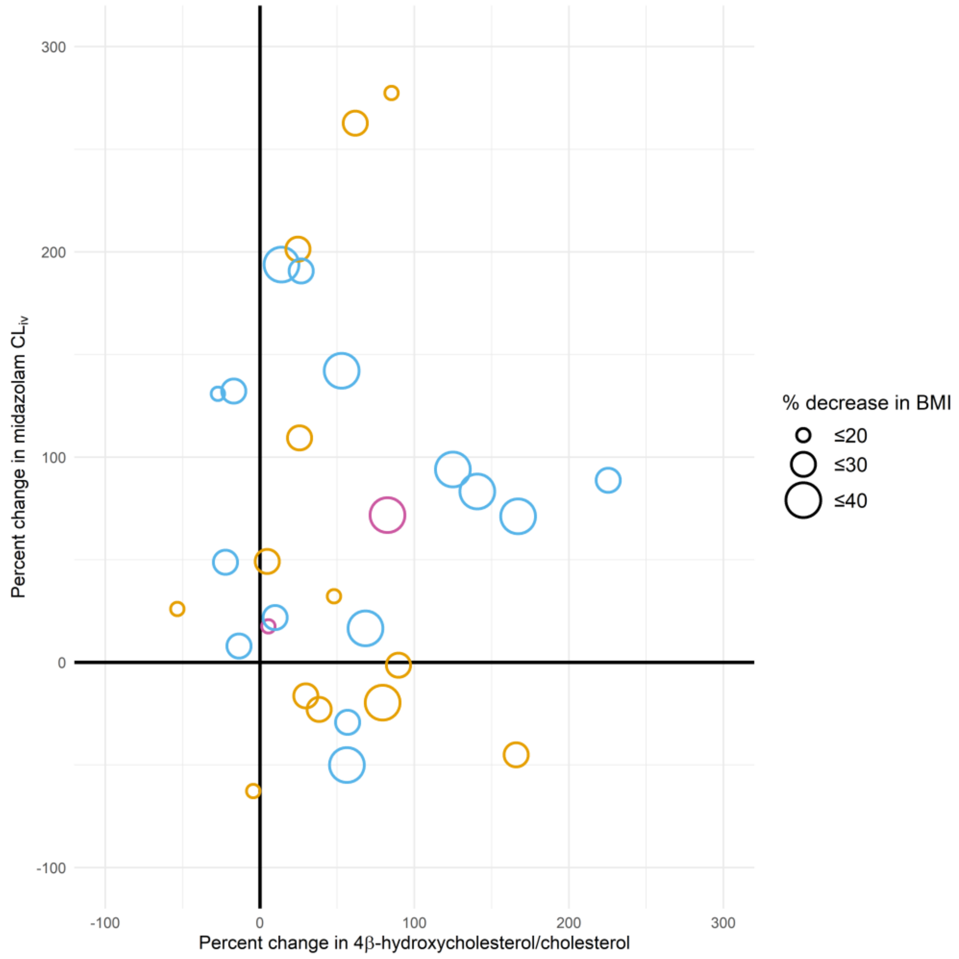


**Figure S3.** Relationship between the percent change in 4β-hydroxycholesterol/cholesterol ratio, and weight-normalised intravenous clearance ($CL_{iv}$) of midazolam at 2-years post-surgery compared to pre-surgery. All data are from the COCKTAIL trial.[1] Each point represents an individual participant, coloured by obesity category: non-obese (blue), obese (orange), and severely obese (magenta) in the year 2 visit. The size of the points reflects the absolute decrease in their body mass-index between pre-and post-surgery.

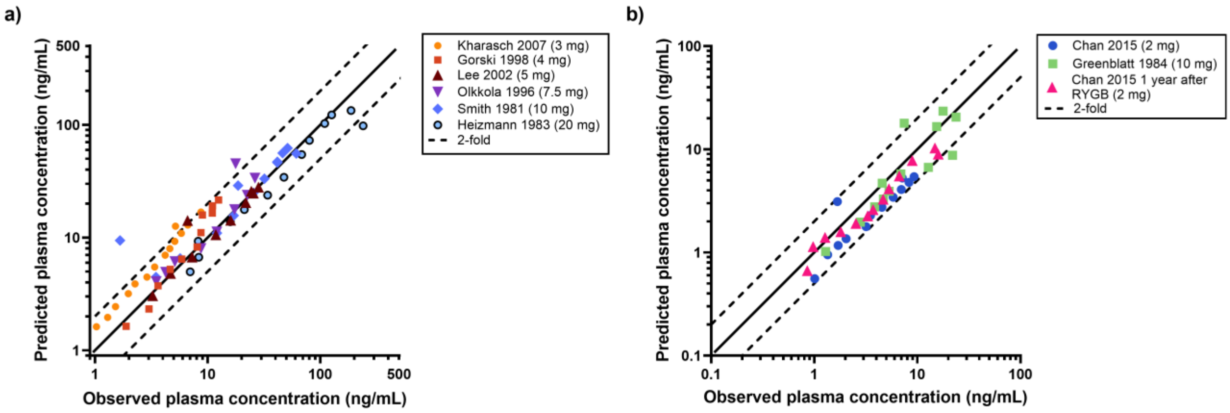


**Figure S4.** Validation of full-body PBPK with ADAM model for oral midazolam by the comparison of predicted and observed plasma concentrations across different dose levels in a) healthy population and b) obese, severely obese and 1 year post RYGB (Roux-Y gastric bypass) surgery. Study details and references are provided in Suppl. Table 3.

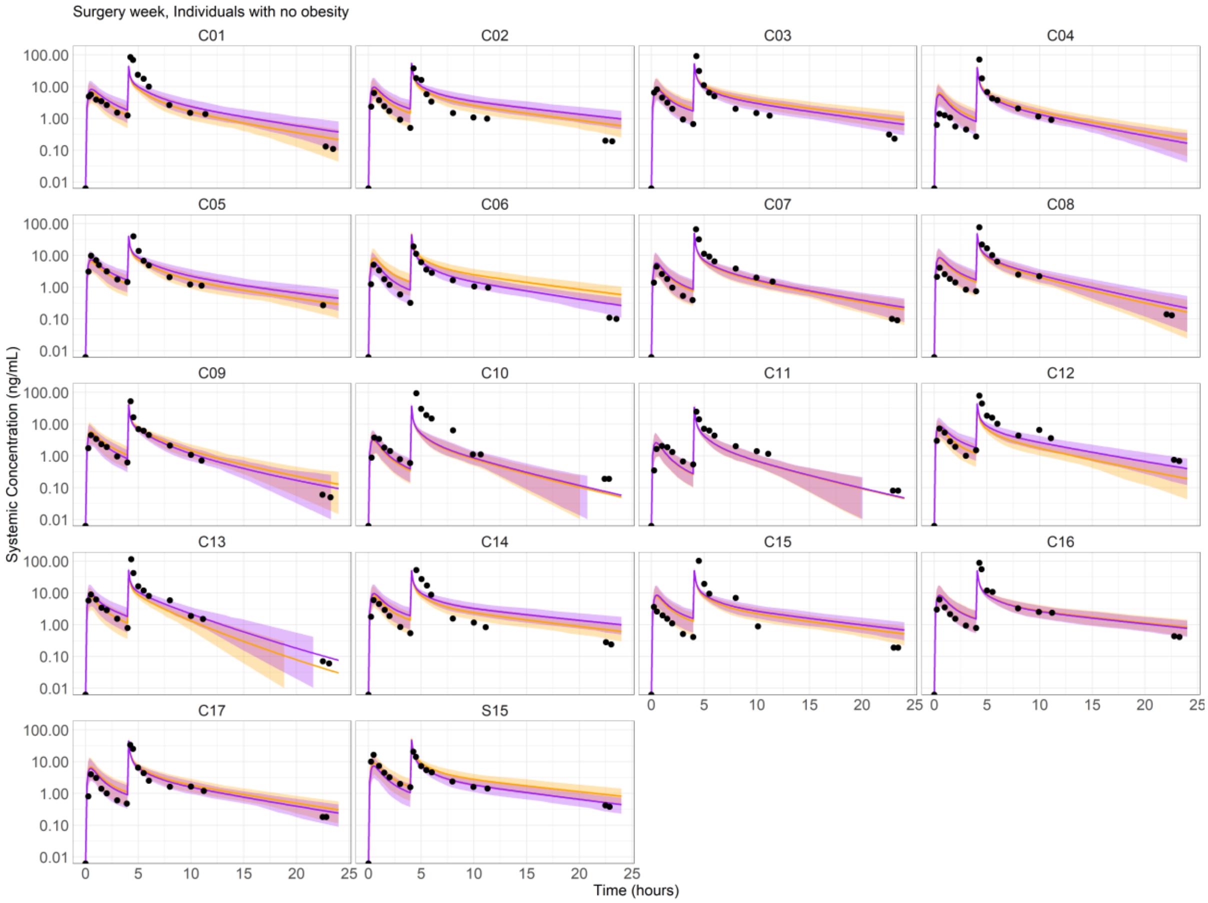


**Figure S5.** Simulation of the midazolam pharmacokinetics of non-obese individuals using the virtual twin PBPK models with either measured (orange) or predicted (purple) CYP3A4 abundance via biomarker approach. Solid lines represent the predicted profiles. Circle symbols represent observed data from the COCKTAIL trial.[1] Areas representing the 5th and 95th percentiles of predictions are shaded with matching colours to predicted lines.

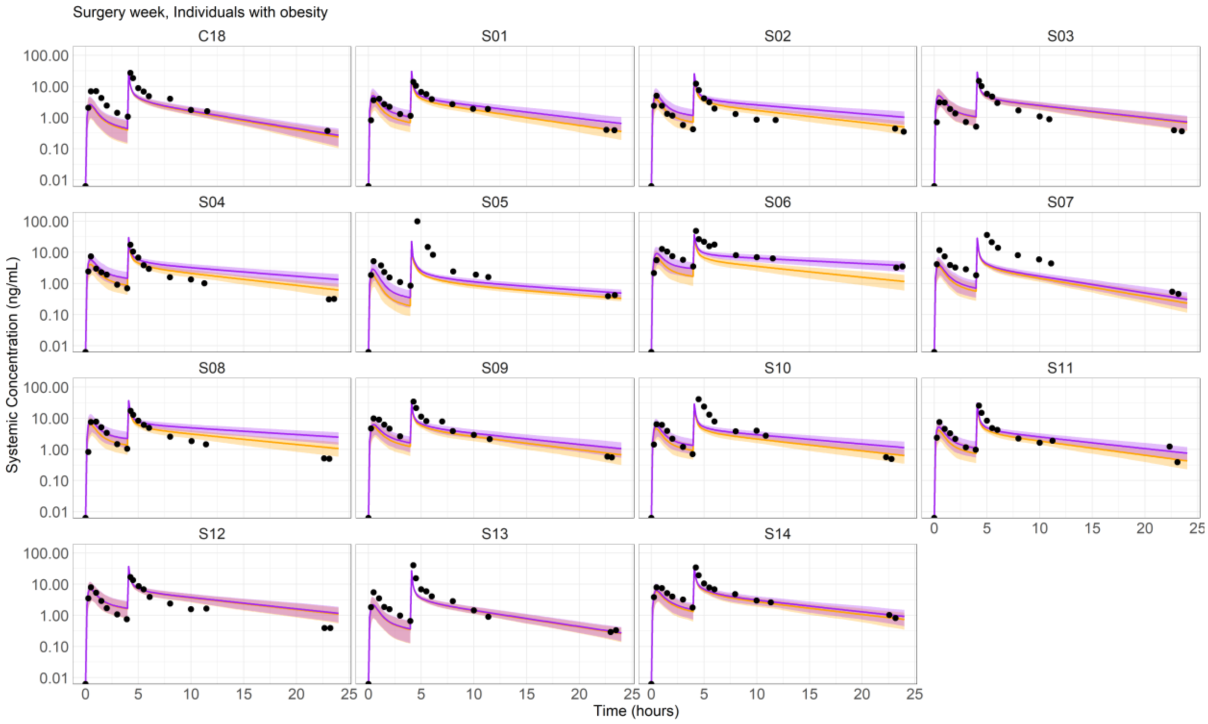


**Figure S6.** Simulation of the midazolam pharmacokinetics in individuals with obesity using the virtual twin PBPK models with either measured (orange) or predicted (purple) CYP3A4 abundance via biomarker approach. Solid lines represent the predicted profiles. Circle symbols represent observed data from the COCKTAIL trial.[1] Areas representing the 5$^{th}$ and 95$^{th}$ percentiles of predictions are shaded with matching colours to predicted lines.

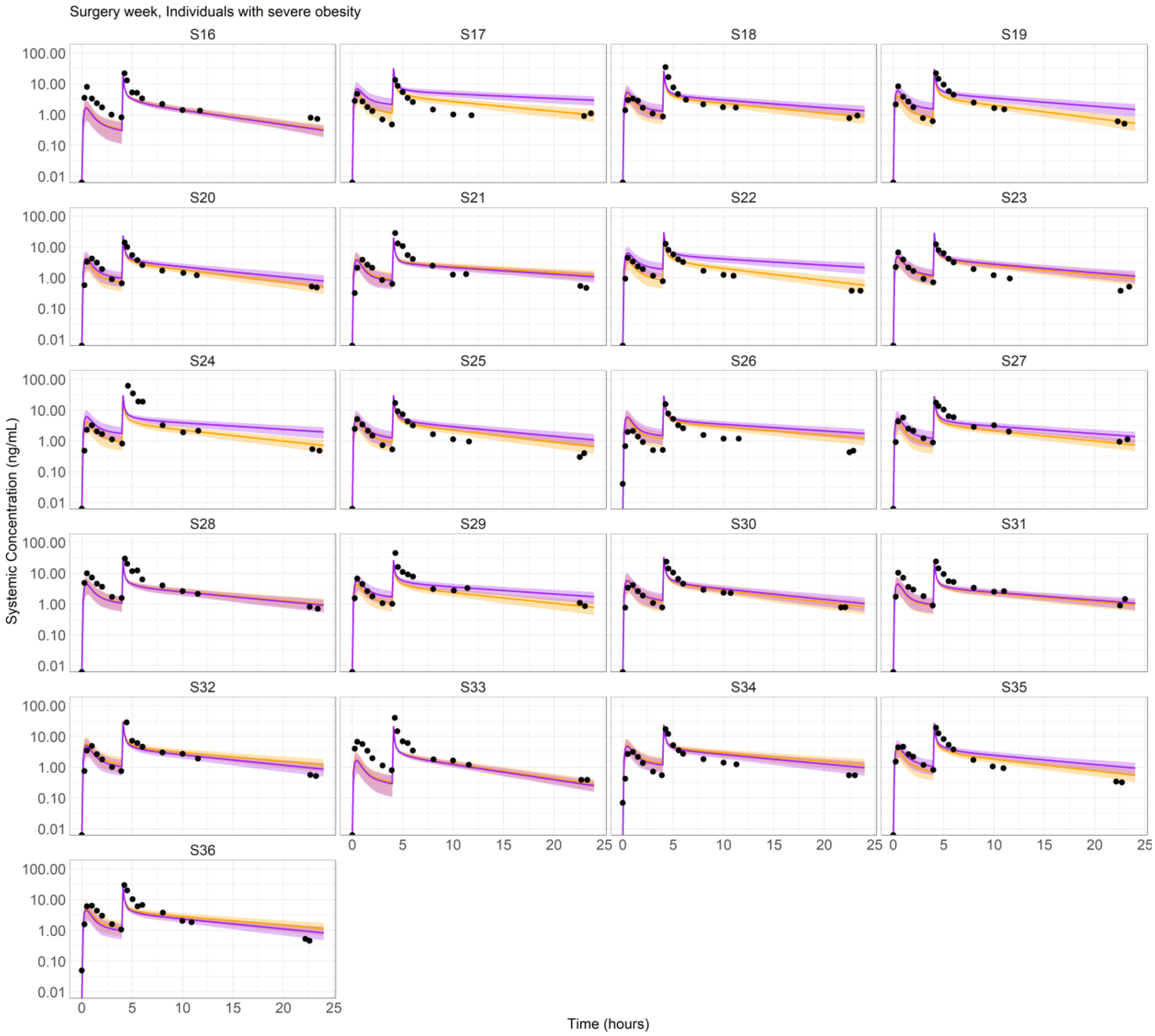


**Figure S7.** Simulation of the midazolam pharmacokinetics in individuals with severe obesity using the virtual twin PBPK models with either measured (orange) or predicted (purple) CYP3A4 abundance via biomarker approach. Solid lines represent the predicted profiles. Circle symbols represent observed data from the COCKTAIL trial.[1] Areas representing the 5th and 95th percentiles of predictions are shaded with matching colours to predicted lines.

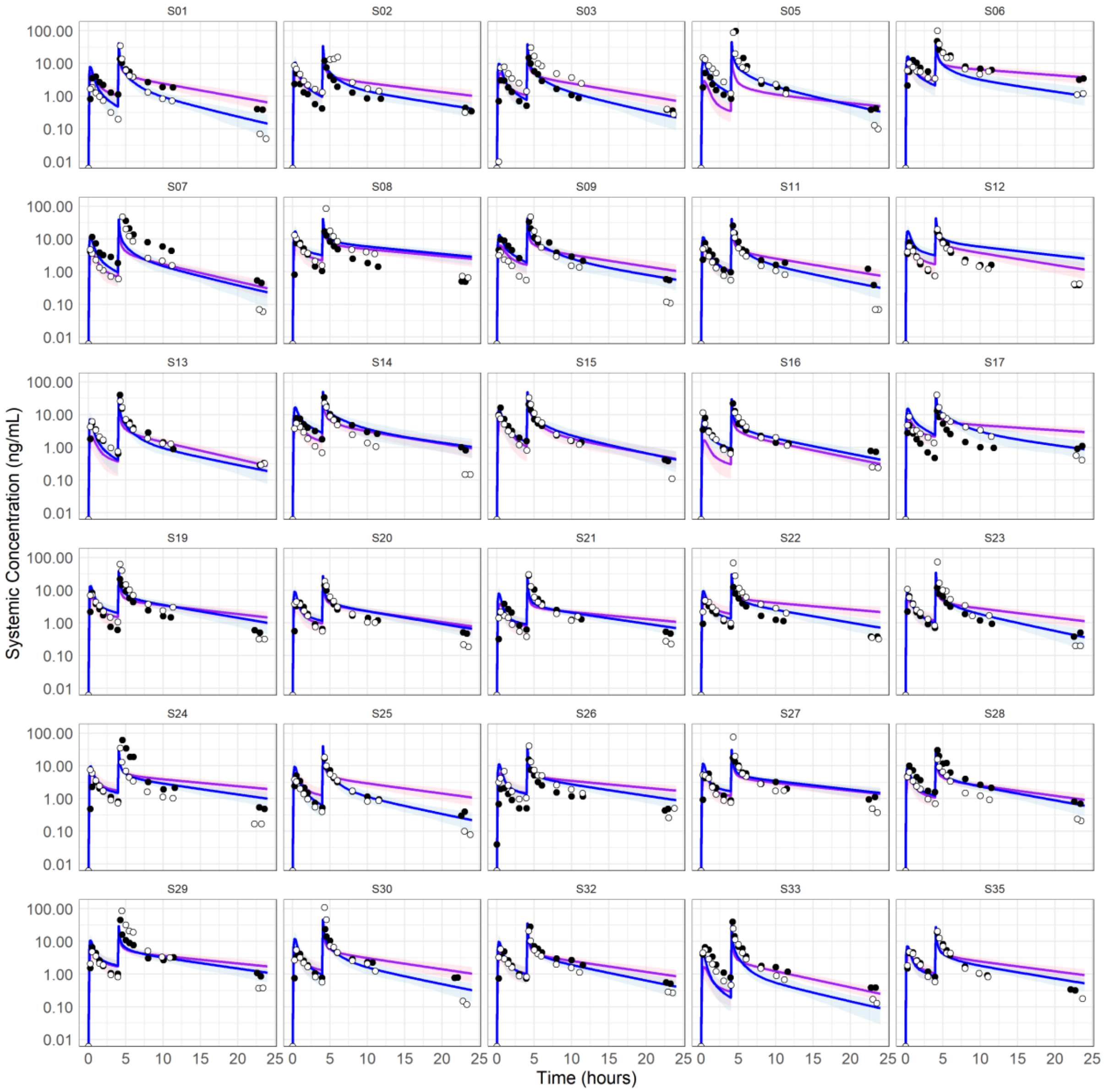


**Figure S8.** Simulation of the midazolam pharmacokinetics using the virtual twin PBPK models using predicted CYP3A4 abundance via biomarker data at the surgery week (purple) and in year 2 (blue). Solid lines represent the predicted profiles. Black and white circle symbols represent observed data from the COCKTAIL trial at the surgery week and in year 2, respectively.[1] Areas representing the 5$^{th}$ and 95$^{th}$ percentiles of predictions are shaded with matching colours to predicted lines.

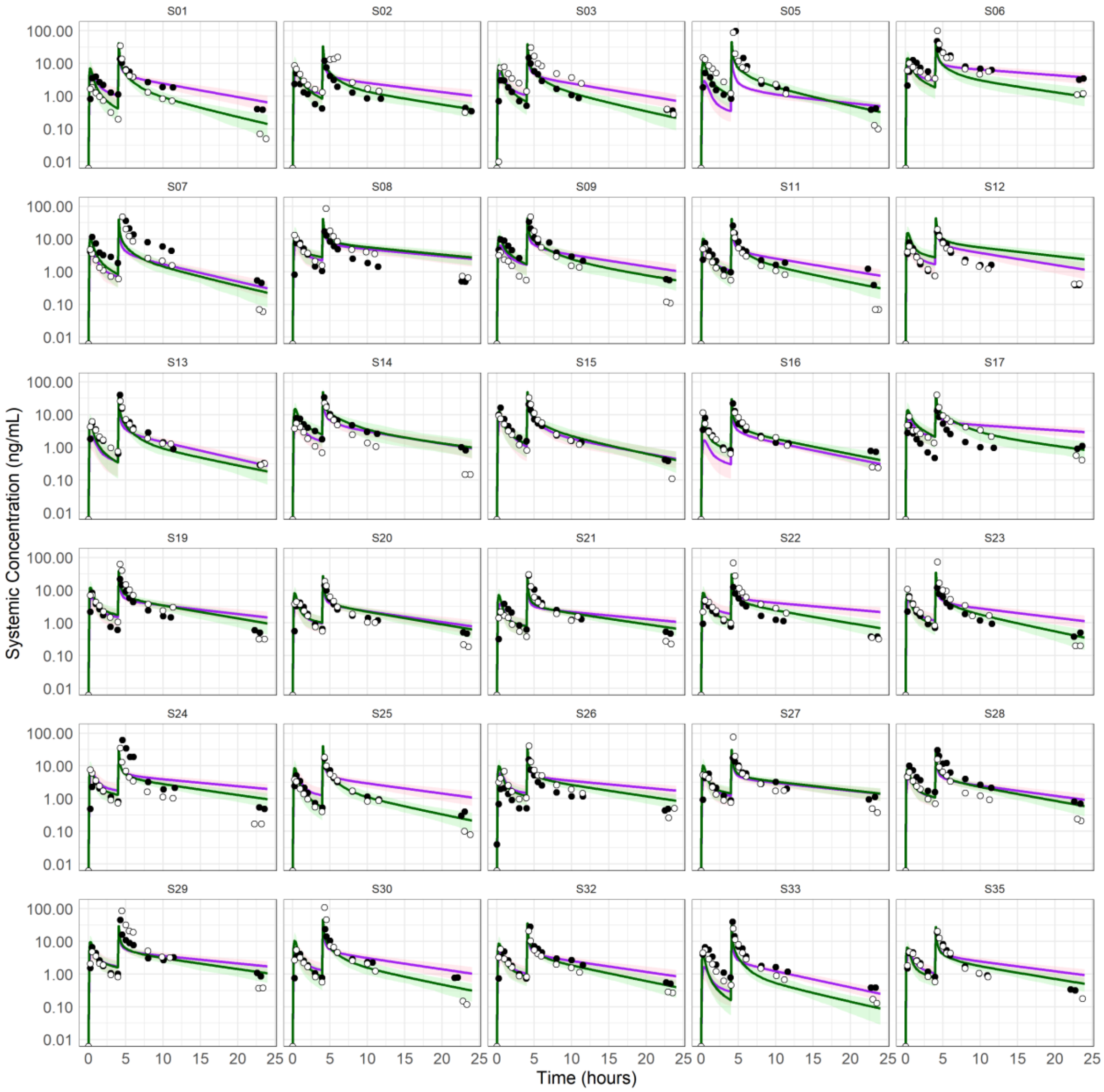


**Figure S9.** Simulation of the midazolam pharmacokinetics using the virtual twin PBPK models using predicted CYP3A4 abundance via biomarker data at the surgery week (purple) and in year 2 with the assumption of full recovery of intestinal CYP3A4/5 (green). Solid lines represent the predicted profiles. Black and white circle symbols represent observed data from the COCKTAIL trial at the surgery week and in year 2, respectively.[1] Areas representing the 5$^{th}$ and 95$^{th}$ percentiles of predictions are shaded with matching colours to predicted lines.

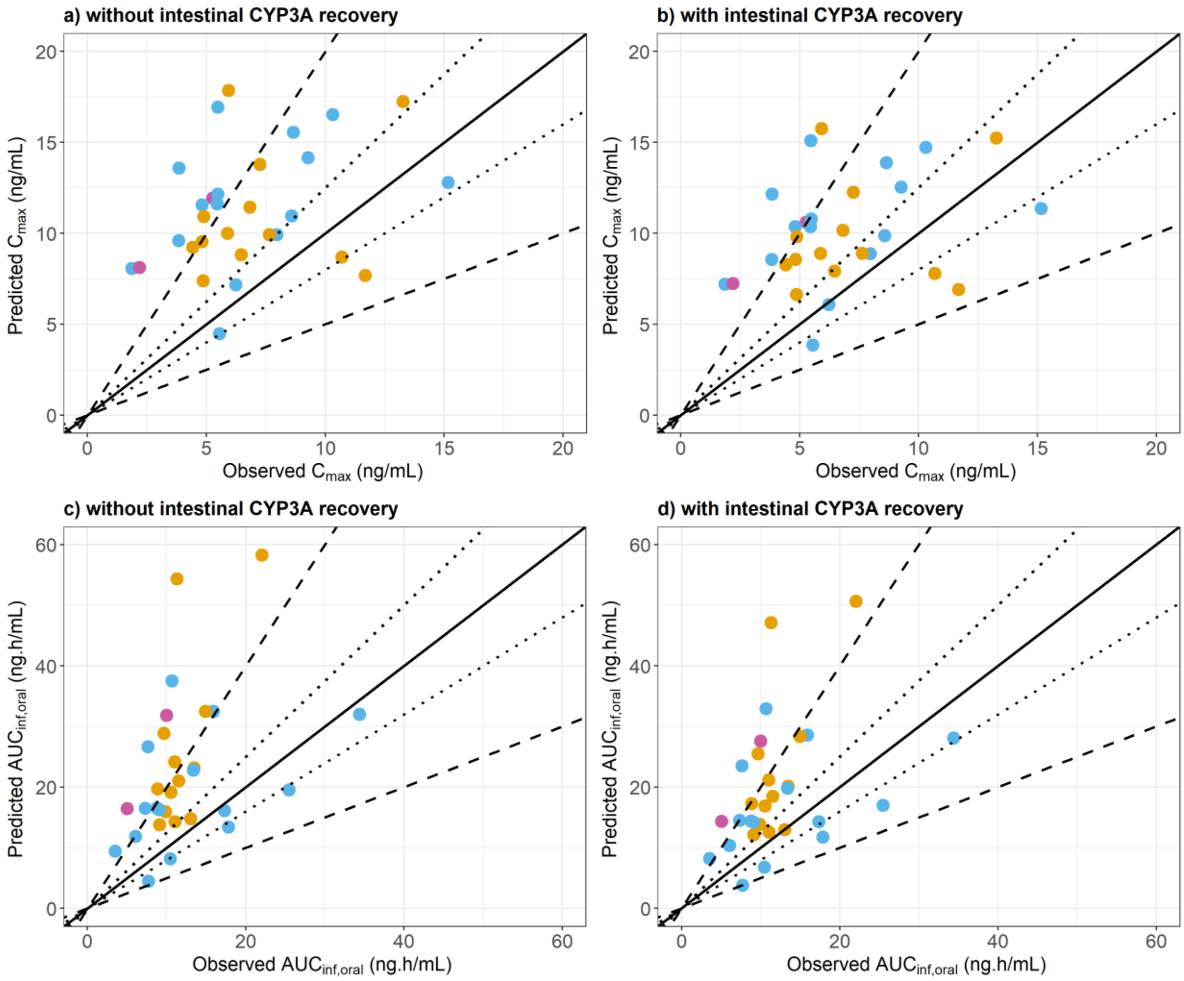


**Figure S10.** Comparison of predicted and observed $C_{max}$ (a, b) and $AUC_{inf,oral}$ (c, d) of oral midazolam 2 years post Roux-Y gastric bypass surgery assuming reduced (a, c) or recovered (b, d) intestinal CYP3A4/5 activity compared to pre-surgery. In each panel, the solid, dotted, and dashed lines represent the line of unity. 1.25-fold difference, and 2-fold difference, respectively. Colours represent obesity status in post-surgery as non-obese (blue), obese (orange), and severely obese (magenta).

## Supplementary References


1. Hjelmesæth J, Åsberg A, Andersson S, et al. Impact of body weight, low energy diet and gastric bypass on drug bioavailability, cardiovascular risk factors and metabolic biomarkers: protocol for an open, non-randomised, three-armed single centre study (COCKTAIL). *BMJ Open*. 2018;8(5):e021878. doi:10.1136/bmjopen-2018-021878

2. Kvitne KE, Hole K, Krogstad V, et al. Correlations between 4beta-hydroxycholesterol and hepatic and intestinal CYP3A4: protein expression, microsomal ex vivo activity, and in vivo activity in patients with a wide body weight range. *Eur J Clin Pharmacol*. 2022;78(8):1289-1299. doi:10.1007/s00228-022-03336-9

3. Kvitne KE, Robertsen I, Skovlund E, et al. Short- and long-term effects of body weight loss following calorie restriction and gastric bypass on CYP3A-activity - a non-randomized three-armed controlled trial. *Clin Transl Sci*. 2022;15(1):221-233. doi:10.1111/cts.13142

4. Wegler C, Wiśniewski JR, Robertsen I, et al. Drug Disposition Protein Quantification in Matched Human Jejunum and Liver From Donors With Obesity. *Clin Pharmacol Ther*. 2022;111(5):1142-1154. doi:10.1002/cpt.2558

5. Wegler C, Matsson P, Krogstad V, et al. Influence of Proteome Profiles and Intracellular Drug Exposure on Differences in CYP Activity in Donor-Matched Human Liver Microsomes and Hepatocytes. *Mol Pharm*. 2021;18(4):1792-1805. doi:10.1021/acs.molpharmaceut.1c00053

6. Kangne H, Izat N, Chen G, et al. Virtual Twin-PBPK Modelling: A Step Toward Precision Dosing in Patients with Obesity. *Aaps j*. 2026;28(1):46. doi:10.1208/s12248-025-01195-7

7. Darwich AS, Pade D, Ammori BJ, Jamei M, Ashcroft DM, Rostami-Hodjegan A. A mechanistic pharmacokinetic model to assess modified oral drug bioavailability post bariatric surgery in morbidly obese patients: interplay between CYP3A gut wall metabolism, permeability and dissolution. *J Pharm Pharmacol*. 2012;64(7):1008-24. doi:10.1111/j.2042-7158.2012.01538.x

8. Kharasch ED, Walker A, Isoherranen N, et al. Influence of CYP3A5 genotype on the pharmacokinetics and pharmacodynamics of the cytochrome P4503A probes alfentanil and midazolam. *Clin Pharmacol Ther*. 2007;82(4):410-26. doi:10.1038/sj.clpt.6100237

9. Gorski JC, Jones DR, Haehner-Daniels BD, Hamman MA, O'Mara EMJ, Hall SD. The contribution of intestinal and hepatic CYP3A to the interaction between midazolam and clarithromycin. *Clin Pharmacol Ther*. 1998;64(2):133-143. doi:10.1016/S0009-9236(98)90146-1

10. Lee JI, Chaves-Gnecco D, Amico JA, Kroboth PD, Wilson JW, Frye RF. Application of semisimultaneous midazolam administration for hepatic and intestinal cytochrome P450 3A phenotyping. *Clin Pharmacol Ther*. 2002;72(6):718-28. doi:10.1067/mcp.2002.129068

11. Olkkola KT, Ahonen J, Neuvonen PJ. The effects of the systemic antimycotics, itraconazole and fluconazole, on the pharmacokinetics and pharmacodynamics of intravenous and oral midazolam. *Anesth Analg*. 1996;82(3):511-516. doi:10.1097/00000539-199603000-00015

12. Smith MT, Eadie MJ, Brophy TO. The pharmacokinetics of midazolam in man. *Eur J Clin Pharmacol*. 1981;19(4):271-278. doi:10.1007/BF00562804

13. Heizmann P, Eckert M, Ziegler WH. Pharmacokinetics and bioavailability of midazolam in man. *Br J Clin Pharmacol*. 1983;16 Suppl 1(Suppl 1):43s-49s. doi:10.1111/j.1365-2125.1983.tb02270.x

14. Greenblatt David J, Abernethy Darrell R, Locniskar A, Harmatz Jerold S, Limjuco Raul A, Shader Richard I. Effect of Age, Gender, and Obesity on Midazolam Kinetics *Anesthesiology*. 1984;61(1)

15. Chan LN, Lin YS, Tay-Sontheimer JC, et al. Proximal Roux-en-Y gastric bypass alters drug absorption pattern but not systemic exposure of CYP3A4 and P-glycoprotein substrates. *Pharmacotherapy*. 2015;35(4):361-9. doi:10.1002/phar.1560

16. Hole K, Heiberg PL, Gjestad C, Mehus LL, Ro O, Molden E. Elevated 4beta-hydroxycholesterol/cholesterol ratio in anorexia nervosa patients. *Pharmacol Res Perspect*. 2018;6(5):e00430. doi:10.1002/prp2.430

17. Lee J, Fallon JK, Smith PC, Jackson KD. Formation of CYP3A-specific metabolites of ibrutinib in vitro is correlated with hepatic CYP3A activity and 4beta-hydroxycholesterol/cholesterol ratio. *Clin Transl Sci*. 2023;16(2):279-291. doi:10.1111/cts.13448

18. Marschall HU, Wagner M, Zollner G, et al. Complementary stimulation of hepatobiliary transport and detoxification systems by rifampicin and ursodeoxycholic acid in humans. *Gastroenterology*. 2005;129(2):476-85. doi:10.1016/j.gastro.2005.05.009

19. Stoch SA, Ballard J, Gibson C, et al. Coadministration of Rifampin Significantly Reduces Odanacatib Concentrations in Healthy Subjects. *J Clin Pharmacol*. 2017;57(1):110-117. doi:10.1002/jcph.780

20. Tomalik-Scharte D, Lutjohann D, Doroshyenko O, Frank D, Jetter A, Fuhr U. Plasma 4beta-hydroxycholesterol: an endogenous CYP3A metric? *Clin Pharmacol Ther*. 2009;86(2):147-53. doi:10.1038/clpt.2009.72
